# Maximizing projects’ profitability, environmental score, and quality: a multi-project scheduling and material ordering problem

**DOI:** 10.1007/s11356-023-26361-2

**Published:** 2023-04-05

**Authors:** Farhad Habibi, Ripon Kumar Chakrabortty, Alireza Abbasi

**Affiliations:** School of Engineering and Information Technology, University of New South Wales, Northcott Drive, Campbell, Canberra, ACT 2600 Australia

**Keywords:** Sustainability, Environmental Impact, Multi-objective optimization, Fuzzy inference system, Project scheduling

## Abstract

The proper trade-off between various project costs is often disregarded when planning projects. This leads to several detrimental effects, such as inaccurate planning and higher total cost, far more significant in a multi-project environment. To overcome this limitation, this study proposes a combined approach for the multi-project scheduling and material ordering problem (MPSMOP), which maintains the proper trade-off among various costs. Moreover, the environmental impact and project quality objectives are optimized alongside the economic criterion. The proposed methodology involves three stages: (a) quantifying the environmental performance of suppliers; (b) measuring the activities’ quality through the Construction Quality Assessment System approach; and (c) building and solving the mathematical model of the MPSMOP. The MPSMOP is modeled as a tri-objective optimization approach aiming to determine project scheduling and material ordering decisions so that the net present value, environmental score, and total quality of implemented projects are maximized simultaneously. As the proposed model comes into the nondeterministic polynomial optimization problem category, two powerful metaheuristics are customized and used to solve the problem. The efficiency of both algorithms was assessed on several datasets. The proposed framework is applied to railway construction projects in Iran as a case study, which presents the validity of the model and the decision-making options provided to managers.

## Introduction

One of the significant concerns of implementing every project is to execute the activities on time under constrained resources. Achieving this goal requires efficient project scheduling. Project scheduling is aimed at developing a detailed plan identifying the activities’ start and finish times, considering the precedence relationships and resource requirements (Lotfi et al. 2022). Various factors can affect the project schedule: inventory management, which includes ordering and storing required stocks in the project sites (RezaHoseini et al. 2021). A broad project schedule is devised based on conventional project planning, followed by developing a material procurement schedule. This strategy disregards the trade-off between inventory costs (including holding and ordering costs) and penalty/reward due to the project’s delayed/early completion time. For illustration, ordering and holding a complete stock of the necessary materials reduce the risk of supply shortages and subsequent inefficiency, such as project delays. However, this approach contributes to a substantial rise in the costs of holding materials on the project site.

On the other hand, materials can be purchased gradually and in small sizes to decrease the holding material costs. Still, there will be risks of their availability and/or delay in delivery. Nevertheless, this strategy increases the likelihood of activity and project delays and the penalty cost (Tabrizi and Ghaderi 2016b). So, it seems vital to simultaneously plan the project scheduling and material supply decisions as this approach can prevent harmful consequences. The detrimental effects of ignoring this trade-off will be much more significant in a multi-project scheduling environment. As an illustration, there will be an increase in total cost relative to the optimal cost when the trade-off between project scheduling and material ordering expenses during the project planning is neglected. So, we have a definite cost difference when planning only one project (if the project is planned according to conventional project planning). The implication is that when we plan, for example, three similar projects simultaneously (a multi-project environment), this cost difference would be higher than in the single-project case.

The project scheduling and material ordering problem (PSMOP) has been introduced and investigated by researchers during the past decades to address this challenge. The integration of project scheduling and material procurement problems was first studied by Aquilano and Smith (1980), where a set of heuristic algorithms was proposed to compute the earliest and latest start times of activities. The preliminary optimization model for the PSMOP was proposed by Smith-Daniels and Smith-Daniels (1987b), who showed that the optimal schedule is obtained only by considering the material ordering plan. In another research item, Smith-Daniels and Smith-Daniels (1987a) stated that the costs and benefits occur over time, and the longer the project finish time is, the more significant the impact of time value will be. Hence, they presented a mathematical model for PSMOP where the system’s net present value (NPV) was maximized concerning material and budget constraints. Dodin and Elimam (2001) investigated an approach that examined the influence of some factors, such as variable duration of activities, variable project value, bonus/penalty due to timely/delayed project completion, and purchasing discounts on the PSMOP. Dodin and Elimam (2008) considered the impact of utilizing special and expensive facilities on project scheduling. They noted that the combination of project scheduling with facility planning provides a novel trade-off leading to a more practical problem and better results.

Sajadieh et al. (2009) improved the model of Dodin and Elimam (2001) in such a way that the ordering times for each activity were determined individually. They proved that the PSMOP comes into the category of NP-hard problems and customized the genetic algorithm (GA) to solve their investigated model on a large-scale problem. After this paper, various methods for solving PSMOP were presented and compared in the literature. For example, Najafi et al. (2011) presented a novel hybrid metaheuristic algorithm where the simulated annealing (SA) algorithm was used for the project scheduling phase, and the GA was utilized for the material ordering stage. Niaki et al. (2015) proposed two-hybrid metaheuristic algorithms, namely, SA-GA and GA-GA. Fu (2014) considered the multi-mode activities in PSMOP to show the dependence of activity duration on resource consumption by selecting different modes of activities. Their research used a hybrid solution technique based on harmonic search (HS) and GA to find near-optimal solutions. Shahsavar et al. (2015) presented an optimization model to investigate the effect of discount policy on purchasing materials in a project scheduling problem and used a hybrid metaheuristic algorithm, namely, GA-PSO, to solve large-scale problems. Tabrizi and Ghaderi (2015a) presented the first multi-objective mathematical model for PSMOP to minimize the project implementation cost and maximize the scheduling robustness. In their model, the discount policy was considered for purchasing materials. Also, they used the *ɛ*-constraint method to determine the Pareto optimal solutions. Despite those efforts, research on PSMOP with more practical details is still an open and challenging research question.

Hence, to bring the problem closer to real-world conditions, many features have been added to the PSMOP. For example, Tabrizi and Ghaderi (2015b) considered the limitation of space availability, and Tabrizi and Ghaderi (2016a) examined considering multiple suppliers for material ordering. Bonus and penalty policy due to timely/delayed project completion time in PSMOP was investigated by Zoraghi et al. (2017). Tayyar et al. (2016a) and Tayar et al. (2016b) investigated the PSMOP in a multi-project but a single-mode environment. Moradi and Shadrokh (2019) presented a model where there were several warehouses with limited storage capacity. Rostami and Bagherpour (2019) studied a multi-period decentralized scheduling problem where a project was managed in decentralized locations. Their primary goal was to determine the optimal schedule, material ordering plan, and resource pool location. They showed that the integrity of decisions could lead to better results. Habibi et al.’s research (2019) was the first research that involved sustainability goals in PSMOP. They proposed a multi-objective mathematical model to determine scheduling and ordering decisions so that the total cost and environmental and social impacts were minimized. To face uncertainties in activity durations and their detrimental effects on the inventory system, Zhang and Cui (2021) presented a two-stage optimization model to obtain robust solutions. Their first stage determined a basic schedule to minimize the total cost, and then, the proactive decisions related to the project schedule and material ordering were made in the second stage. Akhbari (2022) investigated several hybrid metaheuristics, including GA-GA, COA-GA, GWO-GA, and PSO-GA, for PSMOP, considering multi-mode activities and quantity discount policy for ordering materials.

Environmental issues are one of the critical issues of the twenty-first century. Similar to different economic sectors, they are also emphasized in the project management domain, especially when construction projects are considered one of the most important sources of environmental pollution in recent years (Jalaei et al. 2021). Project managers might be willing or forced to achieve better environmental performance and standards since legislation in the environmental area pushed through by the government can affect their decisions when planning projects. By way of illustration, high compensation payments related to weak green performance and environmental damages may be insured against, but this has a high financial burden on companies in the form of insurance premiums (Banihashemi and Khalilzadeh 2021). So, this can motivate the managers to take the trade-off between profit, quality, and environmental practices into account, especially where the prevention cost is far less than the penalty and compensation.

Table 1 summarizes and compares the details of previous research works on PSMOP.

**Table 1:** A summary of PSMOP literature

Paper	Num of objectives		Type of objective(s)					Multiple simultaneous projects scheduling	Characteristic(s)					Resource type		Case study	Solution method
	Single	Multiple	Cost/profit	NPV	Environmental	Quality	Other		Multi-mode	Lead time	Multiple suppliers	Discount offered by multiple suppliers	Penalty and reward	Renewable	Non-renewable		
Dodin and Elimam (2001)	✓		✓							✓			✓		✓		
Dodin and Elimam (2008)	✓		✓										✓	✓			EFL
Sajadieh et al. (2009)	✓		✓							✓			✓		✓		Genetic algorithm
Najafi et al. (2011)	✓		✓												✓		Hybrid method (SA-GA)
Zoraghi et al. (2012)	✓		✓												✓		Hybrid method (SA-GA)
Fu (2014)	✓		✓						✓				✓	✓	✓		Hybrid method (HS-GA)
Niaki et al. (2015)	✓		✓											✓	✓		Hybrid methods (GA-GA, SA-GA)
Tabrizi and Ghaderi (2015a)		✓	✓				✓			✓			✓		✓		E-constraint
Tabrizi and Ghaderi (2015b)	✓		✓							✓			✓		✓		
Shahsavar et al. (2015)	✓		✓											✓	✓		Hybrid method (GA-PSO)
Tabrizi and Ghaderi (2016a)	✓			✓						✓	✓	✓			✓		Genetic algorithm
Tabrizi and Ghaderi (2016b)		✓	✓				✓			✓			✓	✓	✓		NSGA-II
Tayyar et al. (2016a)			✓					✓		✓	✓			✓	✓	✓	
Tayyar et al. (2016b)	✓		✓					✓		✓	✓			✓	✓	✓	Genetic algorithm
Zoraghi et al. (2017)	✓		✓						✓				✓	✓	✓		Hybrid methods (GA-GA, PSO-GA, SA-GA)
Tabrizi (2018)		✓	✓		✓					✓	✓	✓	✓	✓	✓		NSGA-II, MOMBO
Moradi & Shadrokh (2019)	✓		✓							✓	✓				✓		Simulated annealing
Rahman et al. (2019)	✓		✓							✓	✓			✓		✓	Modified MA
Rostami and Bagherpour (2019)	✓		✓											✓	✓		Heuristic methods
Habibi et al. (2019)		✓		✓	✓		✓			✓	✓	✓	✓	✓	✓	✓	NSGA-II, MOPSO, AUGMECON2
Zhang and Cui (2021)	✓		✓												✓		Genetic algorithm
Asadujjaman et al. (2021)	✓			✓						✓	✓			✓			Hybrid method (GA-based MA)
Akhbari (2022)	✓		✓						✓				✓	✓	✓		Hybrid methods (GA-GA, COA-GA, GWO-GA, and PSO-GA)
This Paper		✓		✓	✓	✓		✓	✓	✓	✓	✓	✓	✓	✓	✓	NSGA-II, PESA-II

Although many efforts have been made to develop the PSMOP since 1987, several research gaps still have not been filled in the literature. Table 1 reveals that the following research deficiencies exist:

- The majority of the PSMOP papers have intensely focused on single-objective optimization. However, project managers usually have a set of goals in their minds, so multi-objective optimization is required as a critical tool to optimize their utility function efficiently. Besides, the most considerable amount of work belongs to the simple calculation of cost and profit as objective functions. However, other objectives, such as NPV, quality, system reliability, and environmental effects, have great potential to be considered.
- Investigating PSMOP in a multi-project environment is quite rare. Inconveniently, the detrimental effects of ignoring trade-offs between project scheduling and material ordering expenses will be profound in a multi-project scheduling environment.
- Involving specific features such as multi-mode activities, multiple suppliers, lead time for purchasing materials, discount policy, and bonus/penalty approach can make the problem more realistic. However, these are partially included in existing papers.
- As PSMOP comes into the category of NP-hard problems, there is a need to study new methods to reasonably solve this problem.

As a result, this research addresses the aforementioned research gaps by designing a tri-objective mixed-integer linear programming (MILP) model for the multi-project scheduling and material ordering problem (MPSMOP). This model, with realistic assumptions, is aimed at simultaneously maximizing the NPV, environmental score, and total quality of implemented projects. The scheduling is studied in a multi-project environment with multi-mode activities where the required materials are ordered from multiple suppliers. Two powerful metaheuristic algorithms are customized and used to solve this NP-hard problem. The performance of solution methods is compared using several datasets of different sizes. Eventually, the investigated framework is implemented and validated by a case study on railway construction projects in Iran. Based on Table 1, the crucial contributions of this paper, compared to related research works, are as follows:

- A tri-objective MILP model is presented to maximize simultaneously the total NPV, environmental score, and total quality of the projects. These three objectives have gotten less attention in the related literature. However, legislation in the environmental area pushed through by governments can affect the profit or even quality of projects. This necessitates considering the trade-off between the three aforementioned objectives.
- PSMOP is studied in a multi-project environment often found in the industry. However, almost all of the related papers considered only one single project. Some other critical features, such as procurement time, multiple suppliers, discount strategy, and reward/penalty due to timely or delayed completion time, are considered in this paper.
- Multiple modes for each activity are considered for every single project. As a result, the modes of each activity will differ in duration, quality, consumption of materials, and required renewable resources. This feature is compatible with real-world situations and maintains a better trade-off between the objectives.
- In terms of the solution method, two powerful metaheuristic algorithms, entitled NSGA-II and PESA-II, are customized to solve the model. Although PESA-II has an excellent performance in determining the Pareto optimal solutions, using this algorithm in the PSMOP literature is quite rare.

The remainder of the paper is arranged as follows: the “Methodology” section is devoted to the methodology and describes how the research framework and optimization model evolved. The “Solution process for the MPSMOP model” section of the paper describes the solution process, and its experimental results are discussed in the “Experimental results for the MPSMOP model” section. The “Case study” section is assigned to the information and the outcome of implementing the proposed model in a case study. Eventually, the conclusion and directions for future research are stated in the “Conclusion” section.

## Methodology

In this section, the problem structure and the relevant assumptions are defined, and then, the modeling approach is explained in detail.

### Problem description

**Fig. 1:**
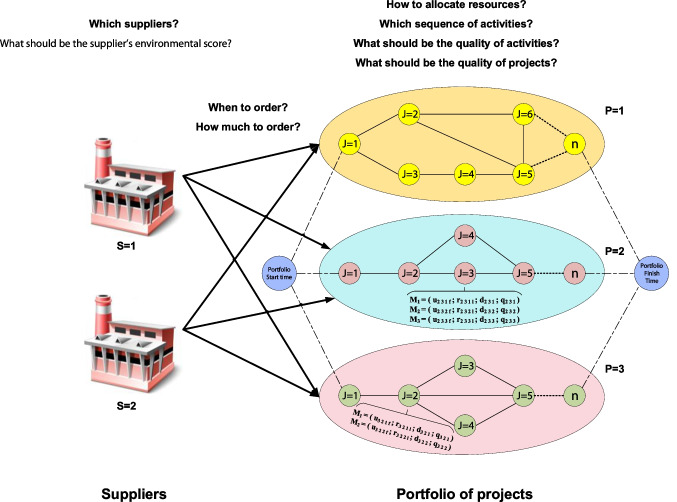
The general framework of the proposed the MPSMOP model

Based on Fig. 1, the proposed framework investigated in this research encompasses a portfolio of projects and several suppliers shown with indices* P* and *S*, respectively. Each project comprises *n* activities to be executed without interruption. The structure of projects is defined by the activity on node structure *G*(*N,A*), in which *ND* and *AC* represent the sets of nodes (activities) and arcs (finish-to-start relationships). As a requirement for AON, two dummy nodes 1 and *n,* with zero processing time and resource requirements, have been used to show the projects’ beginning and finishing points. In all projects, each activity (*J*) can be performed in several modes (*M*) with different renewable and non-renewable resource usages (*r* and *u*), durations (*d*), and qualities (*q*). By way of illustration, consider excavation work as an activity that can be carried out by two modes of automation and non-automation, each of which requires specific renewable (e.g., workforce or machinery) and non-renewable (e.g., required materials) resources with different processing times and implementation qualities.

A fixed quantity of renewable resources is available for performing activities in each period; however, materials should be procured from potential suppliers. Required materials can be purchased from several suppliers, each of which has a specific strategy for the quantity discount as the incentive to order materials in greater numbers/amounts. Resource allocation, supplier selection, ordering time, ordering amount, and scheduling of projects are the major decisions in this system. The proposed mathematical model is aimed at determining these decisions so that the NPV of implementation, environmental score, and total quality of projects are maximized simultaneously.

Additional assumptions include the following:

- Projects are managed decentrally by a single decision-maker. Since projects are geographically far from each other, resources are not shared across projects during implementation, although they can be ordered altogether to take advantage of quantity discounts.
- All required materials for executing each activity should be available before the activity’s start time.
- Preemption in activities is not allowed.
- Each activity can be executed in only one mode (i.e., mode switching is not allowed).
- Materials are consumed equally over the processing time of the activities.
- The all-unit discount policy is considered for material procurement. Based on this policy, the price related to the discount interval is devoted to the entire material units.
- Each project has its penalty and bonus due to the delay and finishing before the due date.

At least two parties participate in real-world projects, including the client (the project owner) and the contractor. According to the contract, payments (positive cash flow) are made in various ways. However, expenses (negative cash flows) are incurred gradually by the contractor during the execution of activities. The most common methods of payments used in real-world circumstances are discussed as follows:

- Lump-sum payment (LSP): in this approach, the contractor receives all its receipts (positive cash flows) at once at the project’s completion time.
- Payments of activities (PAC): according to this strategy, the revenues related to each activity are earned at the finish time of that activity.
- Payments at event occurrences (PEO): based on the PEO model, the positive cash flows are accrued when pre-agreed-upon activities are executed.
- Equal time intervals (ETI): in this system, equal payments are made at regular intervals during the project so that the last one is received once the project is completed.
- Progress payment (PP): according to this approach, receipts are received at specific intervals during project implementation. By way of illustration, positive cash flows can be accrued monthly based on the amount of progress made. Unlike ETI, PP considers the work progress.

The LSP is an ideal payment method for the clients since they pay the contractor once the project is finished. At the same time, this policy imposes a heavy financial burden on the contractors that may not be applicable in some cases. As a result, two parties usually negotiate and agree on the method of payment based on the prevailing conditions (Mika et al. 2005). Since PAC, compared to other approaches, does not create any serious financial problems for the two parties and can meet their interests simultaneously, it is widely used as a reasonable method (Mika et al. 2005; Waligóra 2008). In this paper, the PAC model is considered for calculating the NPV of the projects. So, the payments (positive cash flows) are made directly upon completing each activity, and expenses (negative cash flows) are incurred at the start time of activities.

### Modeling approach for the MPSMOP

The major decisions in the designed problem are divided into two categories:

- Multi-project scheduling decisions involve the allocation of renewable and non-renewable resources to the activities, the sequence of executing activities, and their start time.
- Material procurement decisions include selecting suppliers, ordering time, and quantity.

**Fig. 2:**
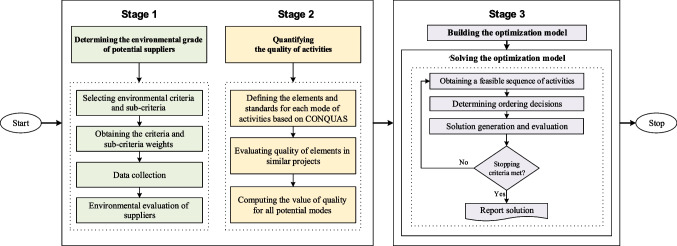
The proposed framework for the MPSMOP with environmental and quality considerations

A methodology based on the tri-objective MILP model is proposed to determine these decisions so that the NPV, environmental grade, and quality of the implemented system are maximized. The proposed framework, illustrated in Fig. 2, consists of three main stages: (a) determining the environmental grade of potential suppliers, (b) quantifying the quality of activities, and (c) building and solving the optimization model.

#### Determining environmental grades of potential suppliers

A fuzzy inference system (FIS), suggested by Habibi et al. (2019), is used to obtain the environmental score of the suppliers. The merit of this approach is to evaluate suppliers’ environmental performance for each type of material. Moreover, the fuzzy logic approach is involved in determining more accurate results. The steps of this approach are as follows:

- Selecting green criteria and metrics: first, several decisive criteria and metrics should be selected for the environmental assessment of suppliers. Hence, a comprehensive list of criteria and metrics was determined by carrying out a review of 16 journal papers (Govindan et al. 2015; Heravi et al. 2015; Kannan et al. 2015; Schöggl et al. 2016; Al-Jebouri et al. 2017; Bottani et al. 2017; Helleno et al. 2017; Kamali and Hewage 2017; Qin et al. 2017; Gupta et al. 2019; Haeri and Rezaei 2019; Liang and Chong 2019; Liu et al. 2019; Gao et al. 2020; Kilic and Yalcin 2020; Rouyendegh et al. 2020). These papers, which thoroughly investigated the green supplier selection (GSS) problem, were extracted from several famous databases, including Elsevier, Emerald insight, Taylor & Francis, MDPI, and Springer. Table 2 summarizes the results.

**Table 2:** Environmental criteria and metrics

Criteria	Metrics
Emission	GHG emission
	Carbon dioxide emission
	Sulphur oxide emission
	Nitrogen oxide emission
Pollution	Sound pollution
Waste	Waste production
	Hazardous waste production
	Wastewater production
	Waste reduction
	Chemical waste production
Resource usage	Water usage
	Energy usage
	Utilizing renewable resources
	The output of used energy
	Paper usage
	Utilizing natural light
Raw material	Utilizing green materials
	Hazardous material consumption
	Recycled material consumption
	Durable material consumption
	Environmental-friendly packaging
	Product waste
	Local material usage
	Packaging waste
Environmental merits	Meeting environmental rules
	Recycling ability
	Durability of products
	Producing recyclable products
	Energy usage monitoring
Reprocessing	Reusing resources
	Recycling waste
	Recycling water
Environmental management system (EMS)	Local monitoring system
	Green management verification
	Invest in environmental development
	Green strategies
	EMS quality

As environmental criteria and metrics can be specific for each organization, a standard process is needed to choose the most relevant and vital indicators from Table 2. The brainstorming technique is used in this research to select suitable indicators. It is shown that this technique can increase harmony among the experts to encourage creativity and refine ideas (Chung and Chung 2019).

- Weighting the environmental criteria: selected criteria and metrics should be compared and weighted due to having different importance. In this regard, multi-attribute decision making (MADM) techniques have an excellent ability to score the selected environmental criteria and metrics (Ning et al. 2016). These techniques mainly use the opinion of experts to rank the most critical and relevant indicators for the system. Some powerful potential MADM techniques are as follows:
- TOPSIS, proposed by Hwang and Yoon (1981), can determine solutions from a set of alternatives by simultaneously minimizing and maximizing the distance from the best (ideal) and worst (nadir) values.
- VIKOR is aimed at ranking the options and choosing the best from definite options, considering the conflicting criteria and proximity to the ideal solution (Sayadi et al. 2009).
- Simple additive weighting (SAW) ranks the alternatives based on their scores, which are calculated as the weighted sum of the attribute (Ravanshadnia et al. 2010).
- Analytic hierarchy process (AHP), introduced by Wind and Saaty (1980), obtains comparisons in pairs of criteria or factors to rank them in each hierarchy stage.
- Analytic network process (ANP) is the generalized version of AHP, where feedback and dependencies among the factors are considered (Görener 2012).

The AHP is the most common technique (Balusa and Gorai 2019). However, AHP loses efficiency when utilized in ambiguous problems like the uncertain nature of criterion parameters. Therefore, the fuzzy AHP technique is used in this paper. This method weighs all environmental criteria through pairwise comparisons by the experts. Readers are referred to Chang (1996) for more information about this technique.

- Collecting data: in this stage, the required data for evaluating the potential suppliers should be collected in terms of the selected criteria and metrics. For example, in the greenhouse gas (GHG) emission metric, the total GHG emitted from the facilities and transport systems (such as used vehicles) will be measured.
- Environmental evaluation of suppliers: first of all, collected data are defined in the form of membership degrees such that they act as the inputs of FIS. Next, the target values of input data, which state the variability interval, are defined as fuzzy numbers. Moreover, the target interval of output variables is converted to [0, 1]. Then, the fuzzy rules that play a critical role in FIS should be described according to the knowledge of experts in the investigated system. Fuzzy rules can obtain the appropriate output through input processing. Finally, crisp values are determined by converting fuzzy values. The environmental grades of supplier *s* for material type *f* can be calculated using Eq. (1).
  1 $${EG}_{fs}=\sum_{h}{\alpha }_{h}{\delta }_{h}$$

where *h* is the index of environmental criteria, *EG* is the environmental grade, ${\alpha }_{h}$ is the weight of the *h*th criterion, and ${\delta }_{h}$ expresses the supplier’s score in the *h*th criterion. As mentioned in the second step, the criteria weights are computed using the fuzzy AHP approach.

#### Quantifying the quality of activities

The modes of every activity have different qualities, and each one can affect the total efficiency of the project. For example, using different building designers and electricians can obtain different qualities from the appearance and electrical installations of the building. In this regard, it is crucial to select a set of modes that can contribute to the maximum quality of the project. However, maximizing quality usually increases the cost and time of project implementation. Therefore, the quality of executable modes can affect the whole project planning, and a critical question is how to evaluate and quantify the quality of activities in different modes. The quality of activities is rarely determined using real data, and most of the current studies in this field deduced their results through hypothetical data (Kannimuthu et al. 2019a). However, there is a wide range of quality evaluation systems to estimate the quality of projects, such as the Performance Assessment Scoring System (PASS) (HKHA, 1994), Quality Assessment System in Construction (QLASSIC) (CIDB 2006), and Construction Quality Assessment System (CONQUAS) (Kannimuthu et al., 2019b). This paper uses a quantification approach based on the CONQUAS approach to estimate the quality of different activities. The CONQUAS approach was selected because it was the basic model for developing other frameworks. In other words, other quality assessment frameworks emerge from the CONQUAS. For example, low-quality construction was common in Hong Kong until the 1980s, and then, the Hong Kong Housing Authority (HA) developed the PASS according to Singapore’s CONQUAS of 1989 (Manap et al. 2017). In addition, CONQUAS consists of three aspects: structural, architectural, and mechanical and electrical (M&E), which make the assessment system compatible with many construction projects (Kannimuthu et al. 2019b). In the CONQUAS approach, three main concepts are stated as follows:

- Item, which means the operation and activity.
- Element, which expresses the subsets and specifications of activity.
- Standard is defined as a list of requirements to be fulfilled by the element.

In order to determine the quality of each mode, several standards are evaluated after the execution of activities. A complete list of these standards can be obtained from the Building and Construction Authority (BCA) of Singapore’s Website.^1^ If all requirements are fulfilled, then that activity’s quality will be 100%. Otherwise, the ratio of fulfilled requirements is considered the value of quality. The quality grade of activities in similar implemented projects must be determined to estimate the expected quality of existing modes. In other words, the quality of activities in similar projects can be used to estimate the expected quality of existing activities. Since each mode of activity has its specific quality, the quality-oriented objective (Eq. (4)) is described as a weighted sum of the minimum and the average qualities of selected activity modes. This objective can maintain the proper trade-off between the average and minimum qualities of the selected modes of activities and maximize the total quality of projects. To put it another way, maximizing the minimum value of quality helps ensure that the selected execution modes of activities in a project do not differ significantly in terms of quality. Besides, maximizing the average quality values of a project enhances the overall quality of that project.

#### Formulating mathematical model for the MPSMOP

The optimization model for the MPSMOP is proposed in the following.

- Indices

*P*: Index of projects; $p\in \left(\mathrm{1,2}\cdots ,P\right)$
*i,j*: Index of activities or operations; $i,j\in \left(1,2,\dots ,{n}_{p}\right)$ that 1 and *n*_*p*_ are dummy operations in project *p*
*m*: Index of modes; $m\in \left(1,2,\dots ,{M}_{pj}\right)$
*l*: Index of renewable resource type; $l\in \left(1,2,\dots ,L\right)$
*f*: Index of material type (non-renewable resources); $f\in \left(1,2,\dots ,F\right)$
*t*: Index of periods; $t\in \left(1,2,\dots ,T\right)$
*s*: Index of suppliers; $s\in \left(1,2,\dots ,S\right)$

- Parameters

*d*_*pjm*_: Duration of activity $j$ of project $p$ performing in mode $m$
*DD*_*p*_: Due date for completing project $p$
*r*_*pjml*_: Number of resource $l$ required periodically to perform activity $j$ of project $p$ in mode $m$
$${R}_{pl}^{\mathrm{max}}$$
Number of available resource type $l$ periodically devoted to project $p$
$${OC}_{fs}$$
Ordering cost of material $f$ which is purchased from supplier $s$
$${HC}_{f}$$
Periodic holding cost of material $f$ per unit
$${PN}_{p}$$
Penalty cost because of delay in completion of project *p* per each period
$${BN}_{p}$$
Bonus because of early completion of project $p$ for each period
$${CF}_{pj}^{+}$$
Positive cash flow because of performing activity $j$ of project $p$
$${CF}_{pjm}^{-}$$
Negative cash flow because of performing activity $j$ of project $p$ in mode $m$
$${EST}_{pj}$$
Earliest start time of activity $j$ in project $p$
$${LST}_{pj}$$
Latest start time of activity $j$ in project $p$
$${PC}_{fks}$$
Procurement cost for ordering one unit of material $f$ in interval $k$ from supplier $s$
$${LT}_{fsp}$$
Lead time that supplier $s$ needs to prepare material $f$ for project $p$
$$Ir$$
Rate of interest
$${\gamma }_{fks}$$
Limitation on discount interval $k$ for material type $f$ related to supplier $s$
$$\left({~}^{P}\!\left/ \!{~}_{F}\right.,Ir\%,t\right)$$
The discount rate which converts future value to the equivalent present value with interest rate $Ir\%$ and time period $t$
$${EG}_{fs}$$
Environmental grade of supplier $s$ for material type $f$
$${q}_{pjm}$$
Quality of activity $j$ executed in mode $m$ related to project $p$
w: Importance weight between the average and minimum qualities

- Decision variables

$${x}_{pjmt}$$
A binary variable which is 1 if activity $j$ of project $p$ is started in mode $m$ in period $t$, 0 otherwise
$${z}_{fkspjmt}$$
A binary variable which is 1 if material $f$ is purchased in interval $k$ from supplier $s$ in period $t$ for activity $j$ of project $p$ performed in mode $m$, 0 otherwise
$${y}_{fkst}$$
A binary variable which is 1 if material $f$ is purchased in interval $k$ from supplier $s$ in period $t$, 0 otherwise
$${I}_{fpt}$$
Level of inventory for material $f$,which is dedicated to project $p$, in period $t$
$${Q}_{p}^{\mathrm{min}}$$
Minimum quality of selected modes in project $p$
$${Q}_{p}^{avg}$$
Average quality of selected modes in project $p$

Model structure

2 $$\begin{array}{c}{\text{M}}{\text{a}}{\text{x}}\;{Z}_{1}=\sum \limits_{p=1}^{P}\sum \limits_{j=1}^{{n}_{p}}\sum_{m=1}^{{M}_{p\;j}}\sum \limits_{t=ES{T}_{p\;j}}^{LS{T}_{p\;j}}C{F}_{p\;j}^{\;+}\;{x}_{p\;j\;m\;t}\left({~}^{P}\!\left/ \!{~}_{F}\right.,\;Ir\%,\;t+{d}_{p\;j\;m}\right)\\ -\sum \limits_{p=1}^{P}\sum \limits_{j=1}^{{n}_{p}}\sum \limits_{m=1}^{{M}_{p\;j}}\sum \limits_{t=ES{T}_{p\;j}}^{LS{T}_{p\;j}}C{F}_{p\;j\;m}^{-}{x}_p\;j\;m\;t\left({~}^{P}\!\left/ \!{~}_{F}\right.,\;Ir\%,\;t\right)\\ -\sum \limits_{f=1}^{F}\sum_{p=1}^{P}\sum \limits_{t=1}^{T-1}H{C}_{f}\;{I}_{f\;p\;t}({~}^{P}\!\left/ \!{~}_{F}\right.,\;Ir\%,\;t)\;-\sum \limits_{f=1}^{F}\sum \limits_{s=1}^{S}\sum_{t=1}^{T}O{C}_{f\;s}\sum \limits_{k=1}^{{K}_{f\;s}}{y}_{f\;k\;s\;t}\left({~}^{P}\!\left/ \!{~}_{F}\right.,\;Ir\%,\;t\right)\\ -\sum \limits_{f=1}^{F}\sum \limits_{s=1}^{S}\sum \limits_{p=1}^{P}\sum \limits_{j=1}^{{n}_{p}}\sum \limits_{m=1}^{{M}_{p\;j}}\sum \limits_{k=1}^{{K}_{f\;s}}\sum \limits_{t=1}^{LS{T}_{p\;{n}_{p}}-L{T}_{f\;s\;p}+1}P{C}_{f\;k\;s}\;{u}_{p\;j\;m\;f}\;{z}_{f\;k\;s\;p\;j\;m\;t}\left({~}^{P}\!\left/ \!{~}_{F}\right.,\;Ir\%,\;t\right)\\ -\sum \limits_{p=1}^{P}\sum \limits_{m=1}^{{M}_{p\;j}}\sum \limits_{t=D{D}_{p}+1}^{T}P{N}_{p}(t-D{D}_{p})\;{x}_{p\;{n}_{p}\;m\;t}\left({~}^{P}\!\left/ \!{~}_{F}\right.,\;Ir\%,\;t\right)\\ +\sum \limits_{p=1}^{P}\sum \limits_{m=1}^{{M}_{p\;j}}\sum \limits_{t=ES{T}_{p\;{n}_{p}}}^{D{D}_{p}-1}B{N}_{p}(D{D}_{p}-t)\;{x}_{p\;{n}_{p}\;m\;t}\left({~}^{P}\!\left/ \!{~}_{F}\right.,\;Ir\%,\;t\right)\end{array}$$

The initial objective function, as Eq. (2), maximizes the NPV of the projects. The first part of the objective is devoted to positive cash flows; the next term states the implementation expenses of projects, including cash outflows, holding costs, ordering costs, and procurement costs. The final part considers the bonus and penalty for completing the projects before and after the pre-determined due dates.

3 $$\text{Max}{Z}_{2}=\sum_{f=1}^{F}\sum_{s=1}^{S}\sum_{p=1}^{P}\sum_{j=1}^{{n}_{p}}\sum_{m=1}^{{M}_{pj}}\sum_{k=1}^{{K}_{fs}}\sum_{t=1}^{LS{T}_{p{n}_{p}}-L{T}_{fsp}+1}E{G}_{fs}{u}_{pjmf}{z}_{fkspjmt}$$

As in Eq. (3), the second objective function maximizes the total environmental score due to suppliers’ material procurement. The environmental grade of suppliers (${EG}_{fs}$) is determined by a fuzzy inference system (FIS).

4 $$\text{Max}{Z}_{3}=\sum_{p=1}^{P}\left[(1-w){Q}_{p}^{avg}+w{Q}_{p}^{\mathrm{min}}\right]$$

Equation (4) is aimed at maximizing the total quality. This objective function mainly involves two parts, as follows:

- The *Max–Sum average quality* is aimed at maximizing the average of the total obtained quality. Although this form of maximizing quality can consider the quality of all activities, it may significantly result in decreasing the quality of one project, because it assumes that the weakness in the quality of one activity can be compensated with the strength of another.
- The *Max–Min quality* is aimed at maximizing the minimum obtained quality. This formulation maintains a balance among the quality of chosen activity modes in all projects. Consequently, there is no significant difference in the quality of projects. In this case, the weakness in the quality of one activity cannot be compensated with the strength of another. However, the total (summation of qualities) may not be at its optimal value.

Equation (4) considers a linear combination between these two formulations to maximize the quality. Using this objective, the main drawback of one part is overcome by the other. The decision-maker will determine the value of *w* to maintain a trade-off between the Max–Sum average and Max–Min quality formulations. To bind the quality criterion between 0 and 1, Eq. (4) can be divided by the ideal point equal to $\left(1-w\right)P+\left(w\right)P=P$.

5 $$\begin{array}{cc}\sum \limits_{m=1}^{{M}_{pj}}\sum \limits_{t=ES{T}_{pi}}^{LS{T}_{pi}}(t+{d}_{pim}){x}_{pimt}\le \sum \limits_{m=1}^{{M}_{pj}}\sum \limits_{t=ES{T}_{pj}}^{LS{T}_{pj}}t{x}_{pjmt}& \begin{array}{c}\forall p=1,2,...,P;\forall j=1,2,...,{n}_{p};\\ \forall i\in Pr(j,p)\end{array}\end{array}$$

Equation (5) defines precedence relationships between the activities of projects.

6 $$\begin{array}{cc}\sum \limits_{j=1}^{{n}_{p}}\sum \limits_{m=1}^{{M}_{pj}}\sum \limits_{{t}^{^{\prime}}=\mathrm{max}(t-{d}_{pjm}+1,ES{T}_{pj})}^{\mathrm{min}(t,LS{T}_{pj})}{r}_{pjml}{x}_{pjm\}{t}^{^{\prime}}}\le {R}_{pl}^{\mathrm{max}}& \begin{array}{c}\forall p=\mathrm{1,2},\dots ,P;\forall l=\mathrm{1,2},\dots ,L;\\ \forall t=\mathrm{1,2},\dots ,{LST}_{p{n}_{p}}\end{array}\end{array}$$

Constraint (6) ensures the availability of renewable resources to start activities.

7 $$\begin{array}{cc}{I}_{fpt}={I}_{fp(t-1)}+\sum \limits_{j=1}^{{n}_{p}}\sum \limits_{m=1}^{{M}_{pj}}\sum \limits_{k=1}^{{K}_{fs}}\sum \limits_{s=1}^{S}{u}_{pjmf}{z}_{fkspjm(t-L{T}_{fsp})}& \forall f=\mathrm{1,2},\dots ,F;\forall p=\mathrm{1,2},\dots ,P;\\ -\sum \limits_{j=1}^{{n}_{p}}\sum \limits_{m=1}^{{M}_{pj}}\sum \limits_{{t}^{^{\prime}}=\mathrm{max}(t-{d}_{pjm}+1,ES{T}_{pj})}^{\mathrm{min}(t,LS{T}_{pj})}\frac{{u}_{pjmf}}{{d}_{pjm}}{x}_{pjm{t}^{^{\prime}}}& \forall t=\mathrm{1,2},\dots ,{LST}_{p{n}_{p}}\end{array}$$

Constraint (7), as the equilibrium equation, calculates the inventory levels of materials at each period.

8 $$\begin{array}{cc}{I}_{fp0}={I}_{fp\left({LST}_{p{n}_{p}}\right)}=0& \forall f=\mathrm{1,2},\dots ,F;\forall p=\mathrm{1,2},\dots ,P\end{array}$$

Constraint (8) expresses that the inventory level of non-renewable resources at the beginning and the end of the planning timeframe is zero.

9 $$\begin{array}{cc}\sum \limits_{m=1}^{{M}_{jp}}\sum \limits_{t={EST}_{pj}}^{{LST}_{pj}}{x}_{pjmt}=1& \forall p=\mathrm{1,2},\dots ,P;\forall j=\mathrm{1,2},\dots ,{n}_{p}\end{array}$$

Equation (9) guarantees that an activity can be executed only once and in one mode.

10 $$\begin{array}{cc}{\gamma }_{f\left(k-1\right)s}{y}_{fkst}\le \sum \limits_{p=1}^{P}\sum \limits_{j=1}^{{n}_{p}}\sum \limits_{m=1}^{{M}_{pj}}{u}_{pjmf}{z}_{fkspjmt}\le {\gamma }_{fks}{y}_{fkst}& \begin{array}{c}\forall f=\mathrm{1,2},\dots ,F;\forall k=\mathrm{1,2},\dots ,{K}_{fs};\\ \forall s=\mathrm{1,2},\dots ,S;\forall t=\mathrm{1,2},\dots ,T\end{array} \end{array}$$

11 $$\begin{array}{cc}\sum \limits_{k=1}^{{K}_{fs}}{y}_{fkst}\le 1& \begin{array}{c}\forall f=\mathrm{1,2},\dots ,F;\forall t=\mathrm{1,2},\dots ,T;\\ \forall s=\mathrm{1,2},\dots ,S\end{array}\end{array}$$

12 $$\begin{array}{cc}\sum \limits_{s=1}^{S}\sum \limits_{k=1}^{{K}_{fs}}\sum \limits_{m=1}^{{M}_{pj}}\sum \limits_{t=1}^{T-1}{z}_{fkspjmt}=1& \begin{array}{c}\forall p=\mathrm{1,2},\dots ,P;\forall j=\mathrm{1,2},\dots ,{n}_{p};\\ \forall f=\mathrm{1,2},\dots ,F\end{array}\end{array}$$

Constraints (10) to (12) are devoted to discounts and procurements. Constraint (10) restricts the quantity of purchased material to the interval between the thresholds of the available discounts. Equation (11) notes that the amount of procured material will come under a limit of one discount range. Equation (12) notes that material procurement is needed to execute activities in all projects.

13 $$\begin{array}{cc}\sum \limits_{s=1}^{S}\sum \limits_{k=1}^{{K}_{fs}}\sum \limits_{m=1}^{{M}_{pj}}\sum \limits_{t=1}^{T-1}\left(t+{LT}_{fsp}\right){z}_{fkspjmt}\le \sum \limits_{m=1}^{{M}_{pj}}\sum \limits_{t={EST}_{pj}}^{{LST}_{pj}}t{x}_{pjmt}& \begin{array}{c}\forall f=\mathrm{1,2},\dots ,F;\forall p=\mathrm{1,2},\dots ,P;\\ \forall j=\mathrm{1,2},\dots ,{n}_{p}\end{array}\end{array}$$

Constraint (13) expresses that every activity can only start after acquiring the required materials.

14 $$\begin{array}{cc}{Q}_{p}^{\mathrm{min}}\le \sum \limits_{m=1}^{{M}_{pj}}\sum \limits_{t={EST}_{pj}}^{{LST}_{pj}}{q}_{pjm}{x}_{pjmt}& \forall p=\mathrm{1,2},\dots ,P;\forall j=\mathrm{2,3},\dots ,{n}_{p}-1\end{array}$$

15 $$\begin{array}{cc}{Q}_{p}^{avg}=\frac{\sum \limits_{j=2}^{{n}_{p}-1}\sum \limits_{m=1}^{{M}_{pj}}\sum \limits_{t={EST}_{pj}}^{{LST}_{pj}}{q}_{pjm}{x}_{pjmt}}{{n}_{p}-2}& \forall p=\mathrm{1,2},\dots ,P\end{array}$$

Equations (14) and (15) calculate the minimum and average values of quality related to selected execution modes, respectively. In other words, Eqs. (14) and (15) determine the values of ${Q}_{p}^{\mathrm{min}}$ and ${Q}_{p}^{avg}$ to be used in Eq. (4) as the third objective function. Please note that the quality values of dummy activities (1 and *n*) are excluded from the set.

16 $$\begin{array}{c}\begin{array}{cc}{x}_{pjmt},{y}_{fkst},{z}_{fkspjmt}\in \left[0,1\right]& \begin{array}{c}\forall j=1,2,...,{n}_{p};\forall p=1,2,...,P;\\ \forall m=1,2,...,{M}_{pj};\forall f=1,2,...,F;\end{array}\\ {I}_{fpt}\ge 0& \begin{array}{c}\forall s=1,2,...,S;\forall t=1,2,...,T;\\ \forall k=1,2,...,{K}_{fs}\end{array}\end{array}\\ \end{array}$$

Finally, Eq. (16) set determines the range of decision variables.

## Solution process for the MPSMOP model

This section explains the solution process, including metaheuristics, solution representation, datasets, evaluation criteria of solution methods, and parameter tuning of the algorithms.

### Solution techniques

Two efficient metaheuristic solution techniques, namely, NSGA-II and PESA-II, are customized to solve the proposed NP-Hard model. It has been proven that NSGA-II yields relatively good results in solving PSMOP (see Habibi et al. 2019). Moreover, although PESA-II can yield better results than NSGA-II in some cases (Gadhvi et al. 2016), the performance of this solution technique on PSMOPs is not investigated yet. Hence, these two algorithms are selected to solve the presented model. NSGA-II was proposed by Deb et al. (2002) to address computational complexity and the non-elitism approach to continuous optimization problems. PESA-II, proposed by Corne et al. (2001), is a multi-objective evolutionary optimization technique that uses the GA method alongside the Pareto envelope-based selection approach. For more information about these two algorithms, the readers are referred to Gadhvi et al. (2016).

Two novel approaches, namely, arithmetic crossover and Gaussian mutation, are used to improve the performance of both algorithms (Furqan et al. 2017; Feng et al. 2018). These two operators can control the algorithms to achieve enough diversity and explore the solution space efficiently when a new population is generated (Eiben and Smith 2015).

Regarding the arithmetic crossover, the parents (${x}_{1}$ and ${x}_{2}$) will be chosen at random, and then, the vector *α* with the same size as the parents will be applied to generate offspring based on Eqs. (17) and (18). This operator linearly combines two chromosomes ${x}_{1}$ and ${x}_{2}$ to provide two offspring ${y}_{1}$ and ${y}_{2}$. The parameter *α* is known as the diversity coefficient.

17 $${y}_{1}=\alpha {x}_{1}+\left(1-\alpha \right){x}_{2}$$

18 $${y}_{2}=\left(1-\alpha \right){x}_{1}+\alpha {x}_{2}$$

Regarding the Gaussian mutation, some chromosomes are picked at random, and then, $\mu$ percent of their genes will undergo a mutation based on Eq. (19). If the values after mutation infringe the defined interval, they will be equal to the corresponding boundary of that interval.

19 $${x}^{^{\prime}}=x+SD\left({\text{Rand}}\left[0,1\right]\right)$$

where *x′* is the mutated gene, *x* is the selected gene, ${\text{Rand}}\left[0,1\right]$ is a random number between 0 and 1, and *SD* is the standard deviation calculated using Eq. (20). This parameter equals a coefficient ($\beta$) of the gene’s variation range.

20 $$SD=\beta \left({Var}_{x}^{\mathrm{max}}-{Var}_{x}^{\mathrm{min}}\right)$$

**Fig. 3:**
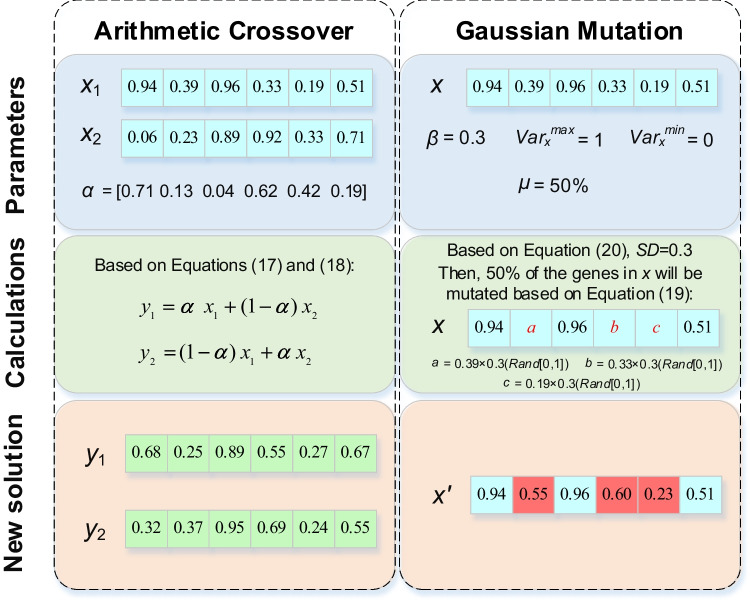
An example to show how operators act

Figure 3 demonstrates an example of how these operators act to provide new solutions.

**Fig. 4:**
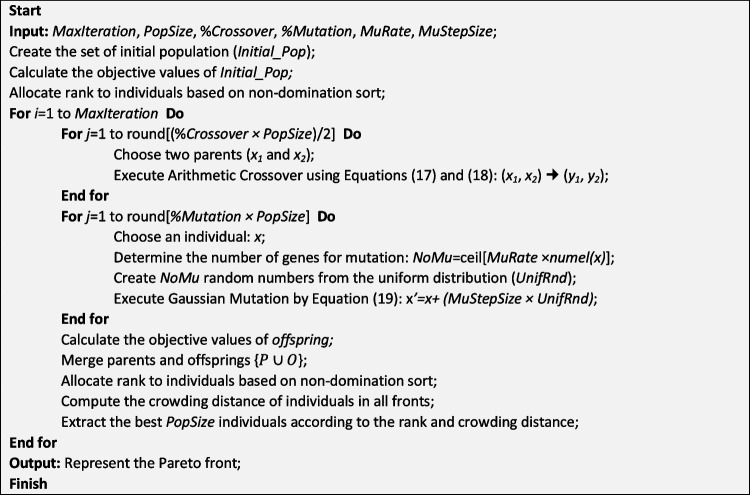
Pseudocode of the customized NSGA-II

**Fig. 5:**
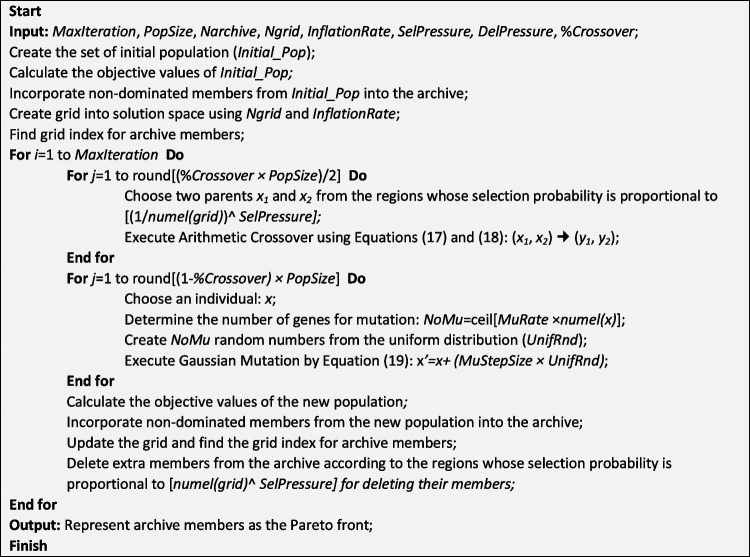
Pseudocode of the customized PESA-II

Figures 4 and 5 represent the pseudocodes of customized NSGA-II and PESA-II for the MPSMOP with the aforementioned improvements.

### Representing solutions

**Fig. 6:**
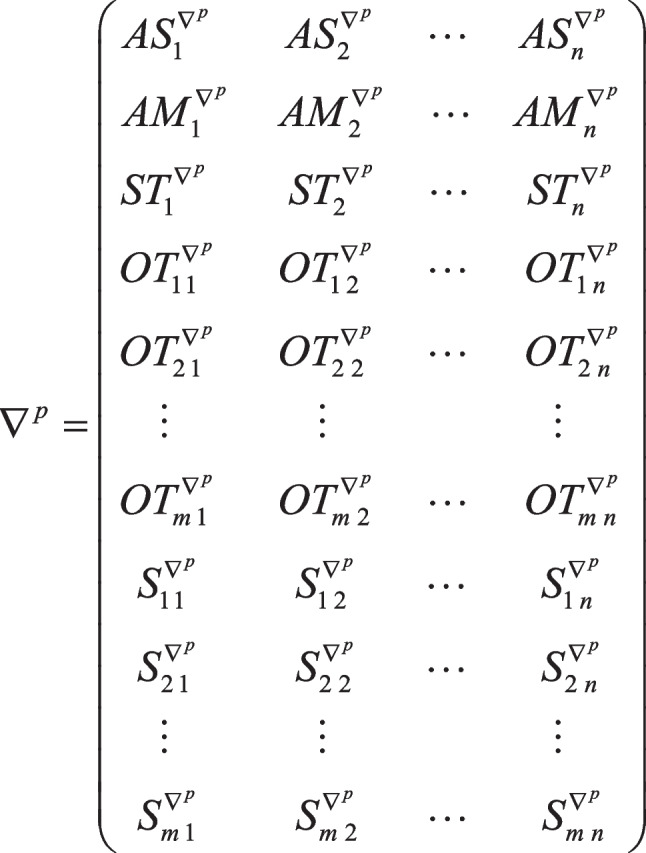
Solution representation of the MPSMOP

Solution representation plays a crucial role in solving models using metaheuristic algorithms. The efficient representation of the solutions assists algorithms in finding the solutions throughout the solution space effectively. In this paper, the set of decisions is demonstrated by the 3D matrix $\nabla$ in Fig. 6. The third dimension of this matrix is devoted to each project. The columns and rows ${\nabla }^{p}$ give information about the activities of the project $p$ and the decisions of each activity, respectively. Regarding the precedence relationships, the feasible sequence of activities (${AS}_{j}^{{\nabla }^{p}}$) is represented in the first row of ${\nabla }^{p}$. The second row is devoted to the selected mode of activities (${AM}_{j}^{{\nabla }^{p}}$). The starting time of activities (${ST}_{j}^{{\nabla }^{p}}$) is shown in the third row of this matrix. The ordering times of materials for each activity (${OT}_{mj}^{{\nabla }^{p}}$) are represented in rows 4 to *m* + 3. Finally, the rows *m* + 4 to 2* m* + 3 represent the selected suppliers from whom the required material is ordered (${S}_{mj}^{{\nabla }^{p}}$).

**Fig. 7:**
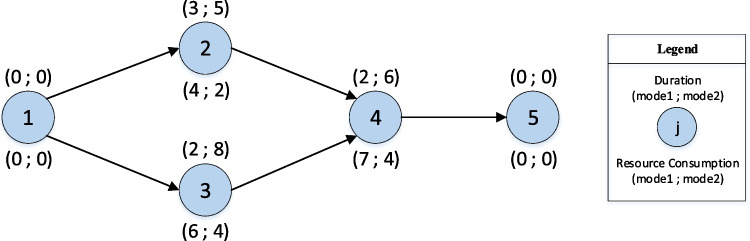
The structure of projects in the example

The following example describes the coding and decoding procedure for solving the MPSMOP. Consider a problem consisting of two projects with five activities, two modes, one non-renewable resource, and two suppliers. Figure 7 represents the structure of both projects and their further details.

As two continuous metaheuristics are investigated to solve the MPSMOP, a novel conversion strategy is used to convert continuous numbers to discrete ones. In this regard, numbers between zero and one are utilized for convenience. Both NSGA-II and PESA-II algorithms can easily work with numbers between zero and one and generate new solutions in each iteration. The following describes how a solution generated by these algorithms is decoded.

**Fig. 8:**
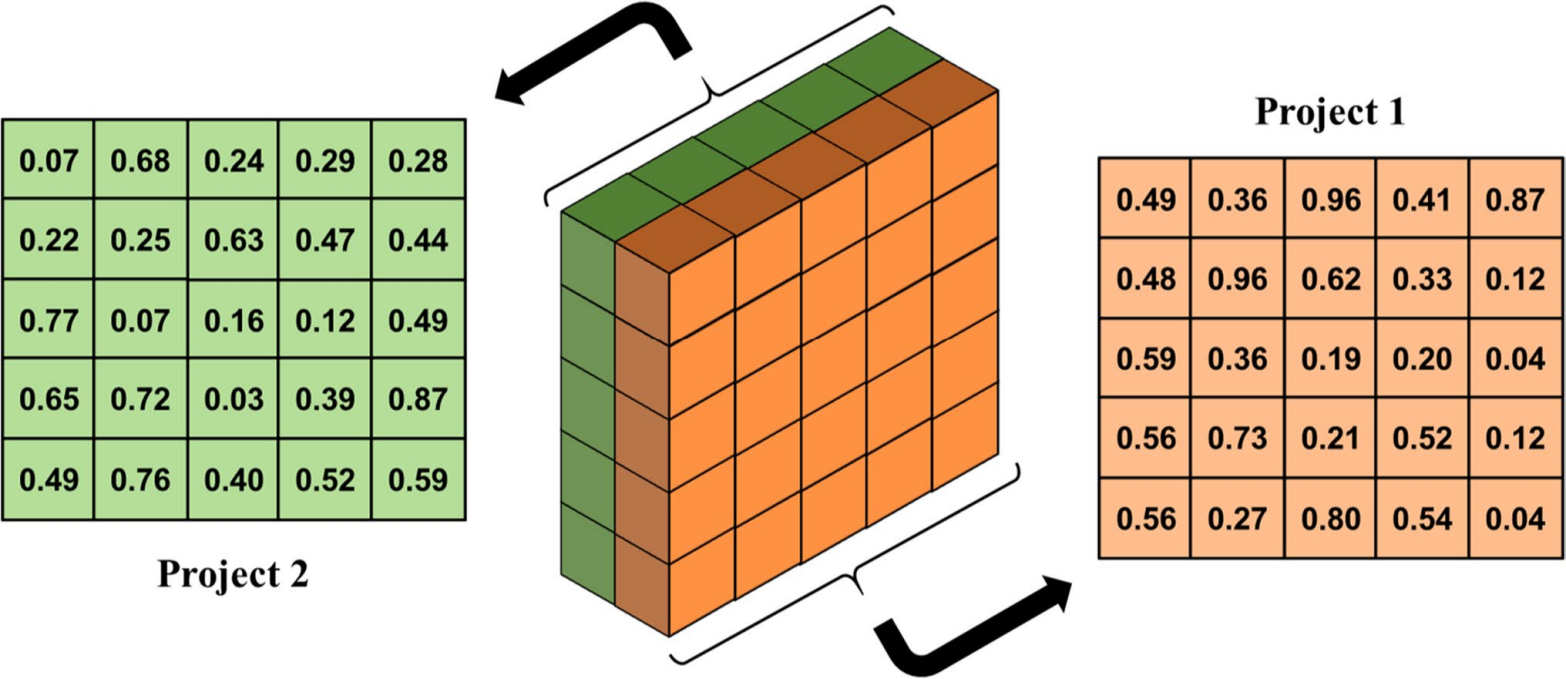
The 3D solution matrix of the example

Given the dimensions of the example and Fig. 6, a 3D matrix shown in Fig. 8 can be interpreted as a solution.

In this three-dimensional matrix, the solutions of the projects are stacked. The solution matrix of Project 1 is illustrated in the following. As mentioned earlier, the first row is devoted to the order of activities. They are converted using the random key strategy, based on which the activities’ permutation is extracted according to the order of the numbers. For example, the first row related to Project 1 is 0.49–0.36–0.96–0.41–0.87, and consequently, the permutation of activities will be 3–1-5–2-4. Since 0.49 is the third smallest number in the string, the number 3 is placed first, and so on. Since it is likely that the order is not feasible in terms of precedence relationship, a repair strategy is used, which rearranges this order to make them feasible. This repair approach puts the first doable activity first and then searches for the next one until all the activities are arranged. So, according to the precedence network in Fig. 7, the infeasible order of 3–1-5–2-4 will be turned into 1–3-2–4-5. For more information about this stage, the readers are referred to Habibi et al. (2017b).

The second row belongs to the modes of activities. Since each activity has two modes, the numbers between 0 and 0.5 indicate mode one, and the numbers between 0.51 and 1 represent mode two. The second row is 0.48–0.96–0.62–0.33–0.12, which shows that the activities should be performed with modes 1–2-2–1-1, respectively.

The third row determines the start time of activities, calculated according to the total floats. Firstly, the critical path method (CPM) calculations should be done, and then, the total floats of all activities are computed. The numbers of the third row will be used in Eq. 21 to determine the start times.

21 $${\text{StartTime}}_{j}={EST}_{j}+{\text{Round}}\left[{ST}_{j}\times {\text{FT}}_{j}\right]$$

where ${EST}_{j}$ is the earliest start time of activity *j*, ${ST}_{j}$ is the number in the solution matrix related to activity *j*, ${\text{FT}}_{j}$ is the float time of activity *j*, and ${\text{Round}}\left[X\right]$ is the function that rounds *X*. So, the start time of activities in Project 1 is 0–1-0–8-10, respectively.

The fourth row of the matrix determines the ordering time of material type one. Since the required material should be ordered in advance, the relevant number in this row (${OT}_{j}$) is multiplied by the difference between the project start time and the start time of that activity, and then, it is rounded. As an example, the start time of activity 4 is equal to 8 and ${OT}_{4}$ is 0.52. So, the ordering time of material type one for activity 4 will be 4.

The last row of the matrix indicates the supplier from whom material type one is ordered. As two suppliers are considered for the problem, the first supplier will be selected if the relevant number in this row is between 0 and 0.5. Otherwise, the orders will be sent to the second one. Therefore, the string of 0.56–0.27–0.80–0.54–0.04 represents that supplier 2–1-2–2-1 will be selected for the activities, respectively. However, there is no need to order materials for the first and last activities as they are dummies.

If the calculations are done similarly for Project two, the results will be shown in Table 3.

**Table 3:** The results of decoding in the example

Details	Activities of Project 1					Activities of Project 2				
	1	2	3	4	5	1	2	3	4	5
Sequence of execution	1	3	2	4	5	1	2	3	4	5
Mode	1	2	2	1	1	1	1	2	1	1
Start time	0	1	0	8	10	0	0	0	8	10
Ordering time	0	1	0	4	1	0	0	0	3	9
Supplier	2	1	2	2	1	1	2	1	2	2

### Test datasets

Several datasets of standard problems in three different sizes are generated and used to evaluate the efficiency of the two metaheuristic algorithms. These three problem sizes consist of problems with 10, 30, and 120 activities. To avoid naming the number of activities, we simply called them small-, medium-, and large-size problems. The parameters of these standard problems are mainly extracted from related literature. For example, for precedence relationships, the project structures are extracted from the project scheduling problem library (PSPLIB). PSPLIB, proposed by Kolisch and Sprecher (1997), is a collection of benchmark instances for testing single- and multi-mode solution procedures for project scheduling problems. Table 4 shows the probability distribution functions of randomly generated datasets and their references. Twenty datasets for each activity and project combinations are created to increase the accuracy of the results. Table 5 gives information about the size and number of various created instances.

**Table 4:** Parameter generation for standard instances

Parameter	Value	Reference/justification
$Pr\left(j\right)$	Extracted from PSPLIB	Kolisch and Sprecher (1997)
${d}_{pjm}$	$\sim {\text{Unif}}\left\{1,10\right\}$	Kolisch and Sprecher (1997)
${DD}_{p}$	Extracted from PSPLIB	Kolisch and Sprecher (1997)
${r}_{pjml}$	$\sim {\text{Unif}}\left\{1,10\right\}$	Kolisch and Sprecher (1997)
${u}_{pjmf}$	$\sim {\text{Unif}}\left\{1,4\right\}$	Tabrizi and Ghaderi (2016a)
${K}_{fs}$	$\sim {\text{Unif}}\left\{1,3\right\}$	Tabrizi and Ghaderi (2016a)
${R}_{pl}^{\mathrm{max}}$	Extracted from PSPLIB	Kolisch and Sprecher (1997)
${OC}_{fs}$	$\sim {\text{Unif}}\left\{5,10\right\}$	Tabrizi and Ghaderi (2016a)
${HC}_{f}$	$\sim {\text{Unif}}\left\{1,5\right\}$	Tabrizi and Ghaderi (2016a)
${PN}_{p}$	$\sim {\text{Unif}}\left\{0,30\right\}$	Kolisch and Sprecher (1997)
${BN}_{p}$	$\sim {\text{Unif}}\left\{0,30\right\}$	Kolisch and Sprecher (1997)
${CF}_{pj}^{+}$	$\sim {\text{Unif}}\left\{5000,6500\right\}$	Tabrizi and Ghaderi (2016a)
${CF}_{pjm}^{-}$	$\sim {\text{Unif}}\left\{60,100\right\}$	Tabrizi and Ghaderi (2016a)
${PC}_{fks}$	$\sim {\text{Unif}}\left\{3,8\right\}$	Tabrizi and Ghaderi (2016a)
${LT}_{fsp}$	$\sim {\text{Unif}}\left\{1,15\right\}$	Tabrizi and Ghaderi (2016a)
${\gamma }_{fks}$	$\sim {\text{Unif}}\left\{5,15\right\}$	Tabrizi and Ghaderi (2016a)
$Ir$	$\sim {\text{Unif}}\left(0.04,0.1\right)$	Tabrizi and Ghaderi (2016a)
${EG}_{fs}$	$\sim {\text{Unif}}\left(0,1\right)$	Parameter nature
${q}_{pjm}$	$\sim {\text{Unif}}\left(0,1\right)$	Parameter nature
$w$	$\sim {\text{Unif}}\left(0,1\right)$	Parameter nature

**Table 5:** The size and number of various created instances

Category	Number of activities	Number of modes	Number of projects	Symbol	Number of created problems
1	10	2	3	J10-M2-P3	20
2	10	2	6	J10-M2-P6	20
3	10	2	9	J10-M2-P9	20
4	30	2	3	J30-M2-P3	20
5	30	2	6	J30-M2-P6	20
6	30	2	9	J30-M2-P9	20
7	120	2	3	J120-M2-P3	20
8	120	2	6	J120-M2-P6	20
9	120	2	9	J120-M2-P9	20
10	10	3	3	J10-M3-P3	20
11	10	3	6	J10-M3-P6	20
12	10	3	9	J10-M3-P9	20
13	30	3	3	J30-M3-P3	20
14	30	3	6	J30-M3-P6	20
15	30	3	9	J30-M3-P9	20
16	120	3	3	J120-M3-P3	20
17	120	3	6	J120-M3-P6	20
18	120	3	9	J120-M3-P9	20
Total number of standard problems					360

Table 5 shows that standard problems have been categorized into different sizes, and 20 problems have been generated for each size. Moreover, the number of renewable and non-renewable resource types and suppliers is considered fixed and equal to 4, 4, and 3, respectively, for all problem sizes.

**Fig. 9:**
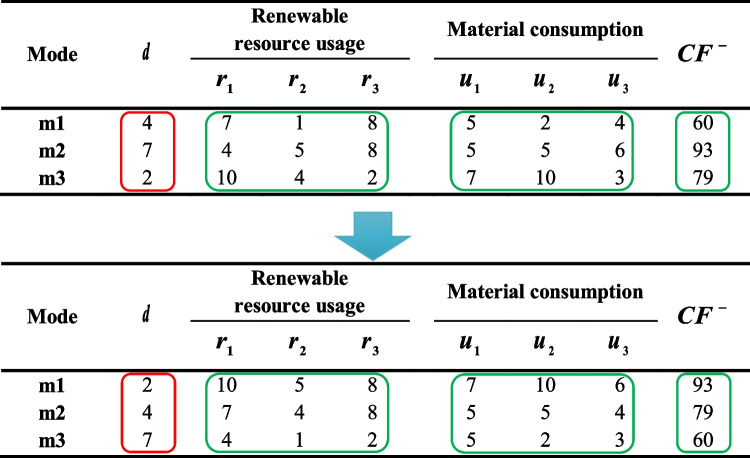
Modifications to the randomly generated data

Besides, some parameters of different modes are modified to obtain a set of non-dominated modes for each activity. These modifications make the problem more realistic as the activity duration can be increased when considering fewer and/or cheaper resources. In other words, a mode with a longer duration has a lower total resource consumption and cost. However, the general structure of the random project and generated random data remain intact. Figure 9 depicts the modification process for an activity. As can be seen, after generating random data, the duration and other parameters (such as resource consumption and cost) are sorted in ascending and descending orders, respectively. Thus, mode 1, the fastest mode, has the highest resource usage and cost, and mode 3 employs cheap resources as the slowest mode.

These self-generated datasets can be obtained from the following link: https://www.unsw.adfa.edu.au/dsar-group/dsarg-datasets. These datasets are categorized with respect to the number of activities (*J*), the number of modes (*M*), and the number of projects (*P*). For example, the file entitled J10-M3-P6 means that this relates to a system that includes 10 activities, 3 modes, and 6 projects. A general guideline for using these datasets is provided in Appendix 2.

### Assessment metrics

To compare and evaluate the solutions obtained from the two metaheuristic algorithms (i.e., NSGA-II and PESA-II), six assessment metrics are considered as follows:

- The number of Pareto solutions (NPS): a higher number of Pareto optimal solutions will contribute to greater flexibility in the decision-making stage. Therefore, the higher the number of NPS, the more efficient the algorithm will be.
- Mean ideal distance (MID): based on Eq. (22), this metric calculates the average proximity of non-dominated solutions from the ideal one (${f}_{1}^{*},{f}_{2}^{*},{f}_{3}^{*}$).
  22 $$MID=\frac{\sum \limits_{j=1}^{NPS}\sqrt{\left(\frac{f_{1j}-f_1^\ast}{f_{1,total}^{\max}-f_{1,total}^{\min}}\right)^2+\left(\frac{f_{2j}-f_2^\ast}{f_{2,total}^{\max}-f_{2,total}^{\min}}\right)^2+\left(\frac{f_{3j}-f_3^\ast}{f_{3,total}^{\max}-f_{3,total}^{\min}}\right)^2}}{NPS}$$
  where ${f}_{ij}$ is the value of the objective *i* in the Pareto solution set and ${f}_{i,{\text{total}}}^{\mathrm{max}}$ and ${f}_{i,{\text{total}}}^{\mathrm{min}}$ are the highest and lowest of these values among all algorithms, respectively. The lower value of the *MID* illustrates the higher efficiency of the algorithm.
- Diversification metric (DM): this criterion represents the extent to which the resulting solutions are distributed in the Pareto front space. As more diverse solutions give the decision-maker a better choice, the higher values of *DM* will be more pleasant. This criterion is calculated using Eq. (23):
  23 $$DM=\sqrt{\left(\frac{\max\{f_{1j}\}-{\min}\{f_{1j}\}}{f_{1,total}^{\max}-f_{1,total}^{\min}}\right)^2+\left(\frac{{\max}\{f_{2j}\}-{\min}\{f_{2j}\}}{f_{2,total}^{\max}-f_{2,total}^{\min}}\right)^2+\left(\frac{{\max}\{f_{3j}\}-{\min}\{f_{3j}\}}{f_{3,total}^{\max}-f_{3,total}^{\min}}\right)^2}$$
- Multi-objective coefficient of variation (MOCV): this metric is essential due to the simultaneous consideration of solutions’ quality and diversification. As Eq. (24) shows, *MOCV* is defined as the ratio between *MID* and *DM*. So, lower *MOCV* demonstrates better performance.
  24 $${\text{MOCV}}=\frac{MID}{DM}$$
- Quality metric (QM): to assess the quality of the results, the total obtained solutions by all metaheuristic algorithms will be compared in pairs. Then, the *QM* of an algorithm is calculated as the proportion of non-dominated solutions of that algorithm. Thus, higher *QM* indicates better efficiency.
- Spacing metric (SM): this criterion measures the regularity of points in the Pareto optimal front and is determined by Eq. (25):
  25 $$SM=\frac{\sum \limits_{j=1}^{NPS-1}\left|{d}_{j}-\overline{d }\right|}{\left(NPS-1\right)\overline{d} }$$
  where ${d}_{j}$ is the Euclidean distance between adjacent solutions, and $\overline{d }$ is the average of these distances. Therefore, the lower the value of *SM*, the more regular the solutions will be in the Pareto optimal front.

### Parameter tuning

Parameter configuration is one of the essential prerequisites to increase the efficiency of finding solutions in metaheuristic algorithms. In this paper, the Taguchi experimental design method proposed by Taguchi et al. (2005) is used for this purpose. This approach can decrease the necessary experiments in a complete factorial experiment so that the desired level of parameters (factors) influencing the solution (response) is identified. In this regard, three levels for each parameter of NSGA-II and PESA-II are defined, and then, the most appropriate level is determined by analyzing the experiments on a medium-size problem (J30-M3-P6). Tables 6 and 7 present the defined levels for each parameter of algorithms.

**Table 6:** Defined parameters for NSGA-II

Factor	Parameter	Levels		
		One	Two	Three
A	Maximum iterations	30	50*	70
B	Size of population	100	150*	200
C	% crossover	0.5	0.7*	0.9
D	% mutation	0.2	0.3*	0.4
E	Rate of mutation	0.18	0.25*	0.32
F	Mutation step size	0.15	0.2*	0.25

*Selected levels to solve the problems

**Table 7:** Defined parameters for PESA-II

Factor	Parameter	Levels		
		One	Two	Three
A	Maximum iterations	50	80*	110
B	Size of population	50	100*	150
C	Size of archive	100	150*	200
D	Number of grids	15	20*	25
E	Inflation rate for grids	0.1	0.15*	0.2
F	Selection pressure	2	4*	6
G	Deletion pressure	6	8*	10
H	% crossover	0.5	0.7*	0.9

*Selected levels to solve the problems

Several indicators, including NPS, MID, DM, SM, and solution time, are considered, and the average of these indicators is designated as the value of the response level to achieve better results. Besides, all these metrics are scaled to the equivalent values between 0 and 100 using the Related Deviation Index (RDI), defined as Eq. 26. This approach makes the data dimensionless before averaging and determining the response level.

26 $$RDI=\frac{\left|\mathrm{Solution}-\mathrm{Best}\;\mathrm{Solution}\right|}{\left|Max\;\mathrm{Solution}-Min\;\mathrm{Solution}\right|}\times100$$

**Fig. 10:**
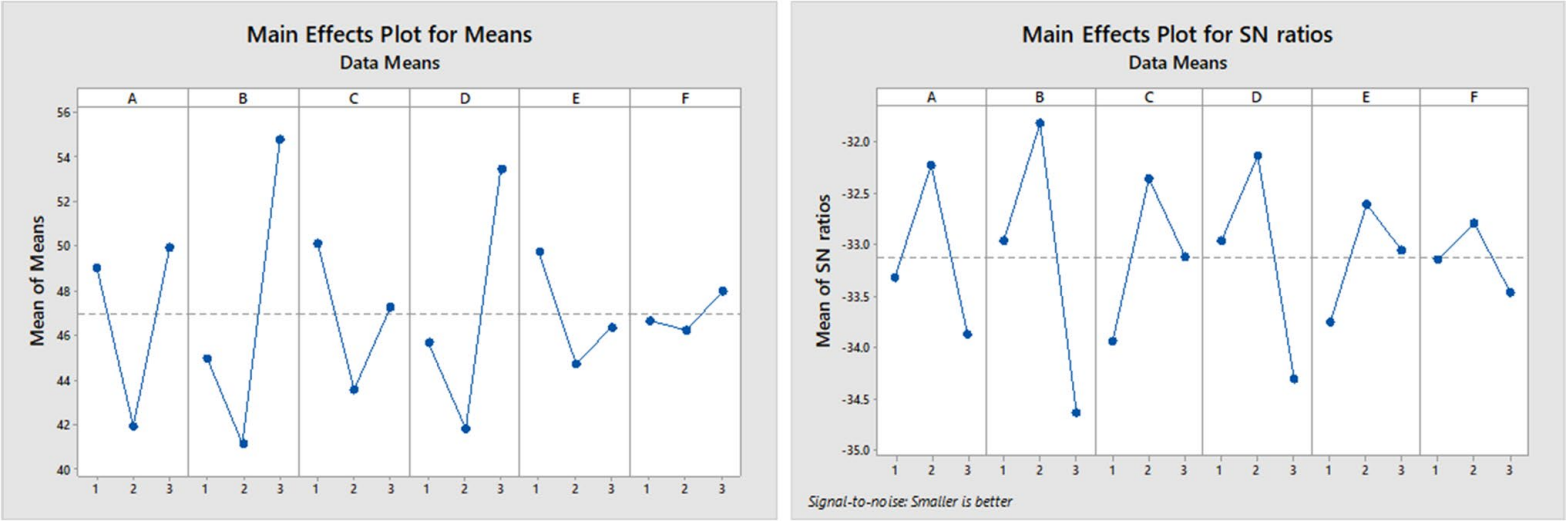
The results of NSGA-II parameter tuning

**Fig. 11:**
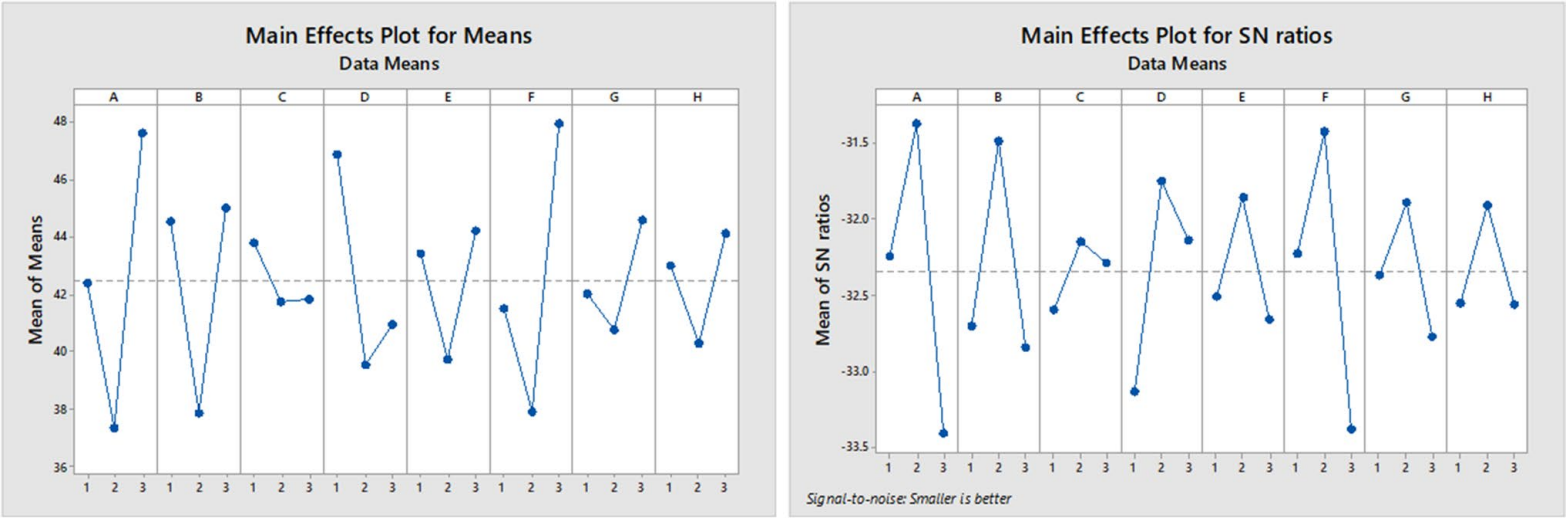
The results of PESA-II parameter tuning

According to Eq. (26), smaller response variable values will be better. The response values of Taguchi DOE for the L27 orthogonal array are given in Table 8. Figures 10 and 11 summarize the outcome of parameter tuning by Minitab software.

**Table 8:** Response values in Taguchi experimental design for parameter tuning

Run No	NSGA-II						PESA-II					
	NPS	Time	MID	DM	SM	Response	NPS	Time	MID	DM	SM	Response
1	75	748.3757	0.8568	1.478	0.0132	**47.45**	43	867.9	1.0235	0.4011	0.0393	**53.24**
2	75	744.733	0.8539	1.4279	0.0121	**46.28**	58	892.1	0.4939	0.6524	0.027	**32.06**
3	90	771.6401	0.9141	1.0009	0.0102	**52.94**	32	879.3	0.8315	0.3443	0.1086	**65.29**
4	80	1646.9	0.9196	1.402	0.0112	**50.62**	57	1721.5	0.492	0.4376	0.0134	**38.12**
5	100	1587.2	0.7628	1.4758	0.0054	**20.88**	98	1701.9	0.4378	0.5280	0.0105	**27.85**
6	99	1599.3	0.8483	1.5509	0.0095	**32.17**	47	1784.0	0.4596	0.524	0.0100	**36.59**
7	70	2718.5	0.9759	1.3329	0.0115	**64.83**	95	2679.1	0.4972	0.4849	0.0117	**34.68**
8	84	2759.2	1.1604	1.0758	0.0114	**75.75**	82	2692.1	0.6547	0.5078	0.0173	**40.42**
9	116	2700.8	1.1704	1.2853	0.0082	**50.53**	70	2699.1	0.8448	0.2186	0.0192	**53.40**
10	100	1927.8	0.6717	1.4662	0.0099	**30.17**	50	1619.6	0.8451	0.9847	0.0076	**31.80**
11	73	1980.8	0.9160	1.5532	0.0119	**51.41**	91	1653.6	0.4632	0.482	0.0378	**35.40**
12	81	2016.8	0.8089	1.438	0.0115	**47.34**	77	1682.1	0.5658	0.2375	0.0318	**44.49**
13	98	2934.8	0.8781	1.3437	0.0071	**41.60**	93	2852.7	0.8072	0.7634	0.0046	**33.98**
14	95	2961.1	0.861	1.3996	0.0062	**38.10**	85	2785.7	0.322	0.2216	0.0132	**39.69**
15	128	2944.4	0.9044	1.4806	0.006	**25.47**	103	2804.3	0.6316	0.4899	0.0092	**36.06**
16	113	2727.3	1.0101	1.259	0.0088	**47.55**	74	4264.3	0.1655	0.2127	0.0003	**42.01**
17	97	2851.3	0.853	1.5683	0.0102	**40.73**	100	4381.5	0.3807	0.2850	0.0165	**44.02**
18	108	2861.4	1.051	1.1459	0.0086	**54.75**	85	4335.6	0.1849	0.8043	0.0119	**29.01**
19	100	2941.7	0.8364	1.2242	0.0086	**46.75**	39	2106.3	0.7397	0.3356	0.0261	**52.24**
20	99	3100.3	0.7773	1.3123	0.008	**41.27**	57	2143.6	1.1583	0.4505	0.0084	**51.99**
21	100	2988.7	0.9155	1.4877	0.0084	**41.20**	63	2096.7	0.2364	0.3213	0.0026	**34.31**
22	101	3513.6	0.9693	1.1855	0.0092	**57.44**	96	4156.6	0.3066	0.3967	0.0085	**38.11**
23	110	3414.9	0.9146	1.2296	0.0065	**43.75**	100	4330.8	0.4807	0.3384	0.0027	**42.01**
24	110	3553	0.8837	1.2449	0.0137	**60.09**	79	4228.1	0.6388	0.3884	0.0111	**48.43**
25	103	4560.4	0.8906	1.0793	0.0089	**61.79**	10	5794.9	0.4155	0.1457	0.0112	**66.79**
26	106	4420.7	0.9419	1.4444	0.0065	**44.54**	138	5675.4	0.4587	0.1980	0.0023	**44.29**
27	129	4481.4	0.8597	0.9479	0.0078	**52.91**	100	5859.1	0.4959	0.3846	0.0202	**50.57**

The lower values of the “mean of means” (as the first criterion) and the higher “SN ratio” (as the second criterion) show better results. Since a three-point analysis is performed, observing any increasing or decreasing trend in these two criteria raises the possibility that higher or lower levels (which have not been analyzed) may be more appropriate parameters than the existing ones. To prevent this, we tuned the parameter a couple of times. This means that after observing the trend in the criteria, we changed the parameter levels and did the Taguchi DOE method to calibrate the parameters. This process continued until the appropriate levels were placed at level 2 (to ensure that suitable parameters were selected). So, that is why we observe that all second levels have the lowest mean of means and the highest SN ratio.

## Experimental results for the MPSMOP model

In the following, the results of solving the MPSMOP model are discussed. First, the solution algorithms are compared with respect to the evaluation criteria, and then, the results of sensitivity analysis on the model parameters are described.

### Performance metrics for comparison

**Fig. 12:**
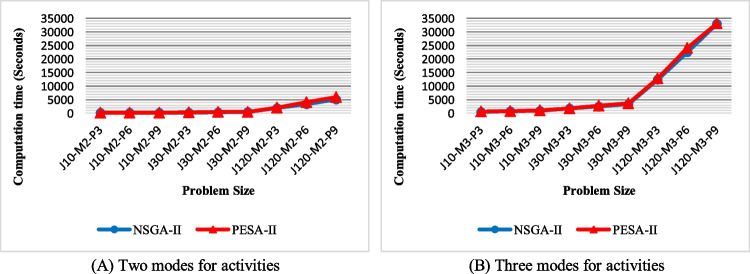
The efficiency of the algorithms with regard to solution time
- The number of Pareto solutions (NPS): regarding this metric, Fig. 13 represents the NPS achieved by solution techniques for the various problem sizes. This figure shows that NSGA-II obtains more Pareto optimal solutions and performs better in terms of NPS. Also, the difference in superiority increases with increasing the size of the problem. There is a sudden drop in the number of Pareto solutions for J120-M2-P3 and J120-M3-P3. This is because the number of projects decreases by changing the problem from J30-M2-P9 and J30-M2-P9 to J120-M3-P3 and J120-M3-P3. Consequently, the system’s decisions and the congestion of solutions in the Pareto optimal set also decrease. Although the number of activities has increased, changing the number of projects has a more significant effect on the number of decision variables. However, NPS is not affected by the number of modes, when comparing Fig. 13A and B.

In this section, the performance of NSGA-II and PESA-II is assessed from the perspective of the mentioned evaluation criteria. The presented model was programmed using MATLAB 8.5 and was run on a computer with Windows 7 (64-bit) OS, Core i7 (2.0 GHz) CPU, and 8 GB RAM. The average of 20 datasets is calculated and evaluated for each problem size to achieve more accurate results. The following evaluation criteria (performance metrics) assess the efficiency of the proposed methods:

- Elapsed time: Fig. 12 shows the computational time of NSGA-II and PESA-II for solving different problem sizes. As can be seen, the solution time of both algorithms increases as the problem size grows. However, PESA-II needs a little more computational time, regardless of problem size. Also, assuming the number of activities is constant, the solution time will increase linearly with increasing the number of projects. The number of modes also impacts the computational time since there is a considerable difference between Fig. 12A and B, especially when the number of activities and projects increases.

**Fig. 13:**
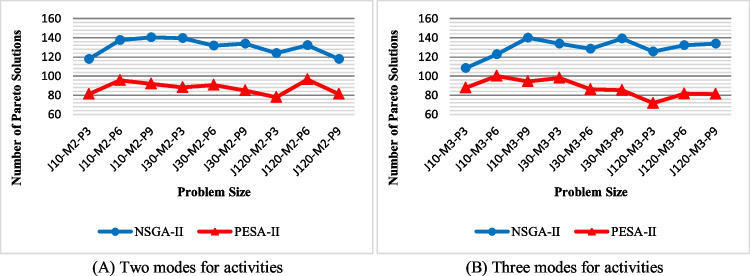
The efficiency of the algorithms with regard to NPS
- Mean ideal distance (MID): Fig. 14 shows the values of MID obtained by the solution techniques. Based on this figure, the PESA-II’s solutions are closer to the ideal solution for all problem sizes. They keep an almost constant distance as a straight line so that they are, on average, 0.447 away from the ideal solution to all problems. Although the solutions of NSGA-II get closer to the ideal solution as the problem size grows, PESA-II still maintains its superiority. Although the number of modes does not impact PESA-II performance, NSGA-II determined closer solutions to the ideal point in problems, including two modes compared to those with three activity modes.

**Fig. 14:**
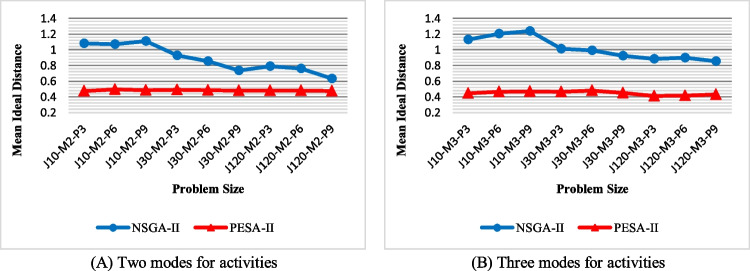
The efficiency of the algorithms with regard to MID
- Diversification metric (DM): the outcome of comparing the two algorithms with regard to DM is shown in Fig. 15. As can be seen, there is no absolute superiority for both algorithms in terms of DM. For small-size problems, PESA-II, and for medium-size and large-size problems, NSGA-II yields more diverse solutions. Figure 15A and B reveals the same trends for problems with two and three activity modes.

**Fig. 15:**
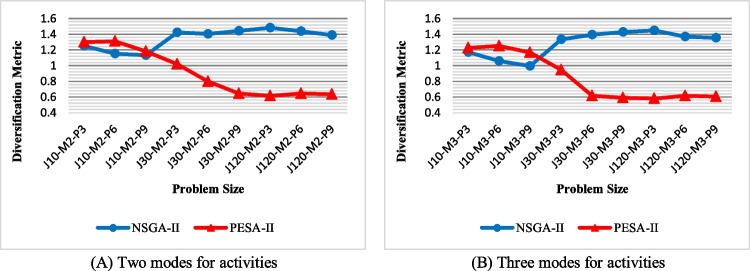
The efficiency of the algorithms with regard to DM
- Multi-objective coefficient of variation (MOCV): as shown in Fig. 16, PESA-II performs better in the four smaller problem sizes in terms of *MOCV*, regardless of activity modes. However, NSGA-II is superior in larger cases. This can be justified by the behavior of NSGA-II in terms of *DM*, because with the increasing problem size, the *DM* for NSGA-II increases, which leads to the superiority of this algorithm from the *MOCV* perspective in large-size problems. For a similar reason, PESA-II has an uptrend.

**Fig. 16:**
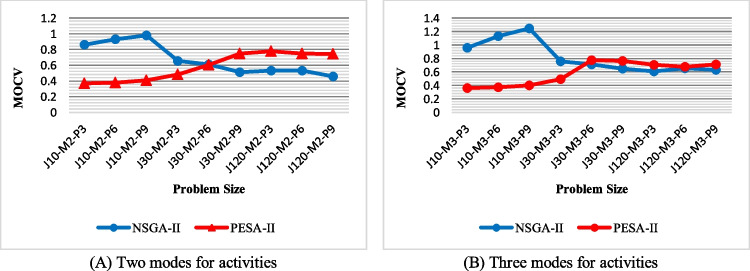
The efficiency of the algorithms with regard to MOCV
- Quality metric (QM): the values of QM achieved by NSGA-II and PESA-II are shown in Fig. 17. As this figure shows, the solution of PESA-II can dominate almost all of NSGA-II’s solutions in small-size problems. The solutions of NSGA-II get higher quality by increasing the size of the problem, because as the problem size increases, the solution space becomes wider, and NSGA-II obtains more diverse solutions not to be dominated by PESA-II. NSGA-II provides higher-quality solutions for problems with two activity modes than those with three modes, although PESA-II still has obtained better solutions in terms of QM for all problem sizes.

**Fig. 17:**
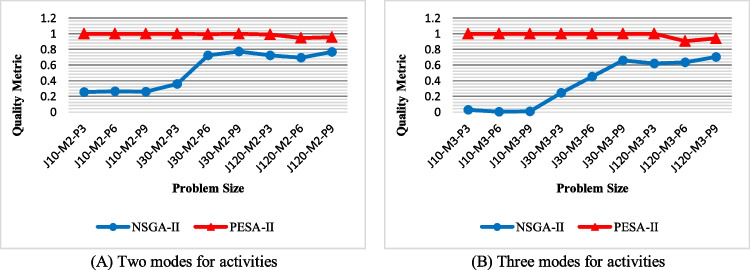
The efficiency of the algorithms with regard to QM
- Spacing metric (SM): the values of SM obtained by NSGA-II and PESA-II are compared in Fig. 18. This figure shows that NSGA-II produces a more regular Pareto optimal front due to having lower SM. Also, the regularity of the solution determined by PESA-II decreases as the problem size grows. This decrease is experienced in problems with two activity modes with a lag when the number of activities and projects increases.

**Fig. 18:**
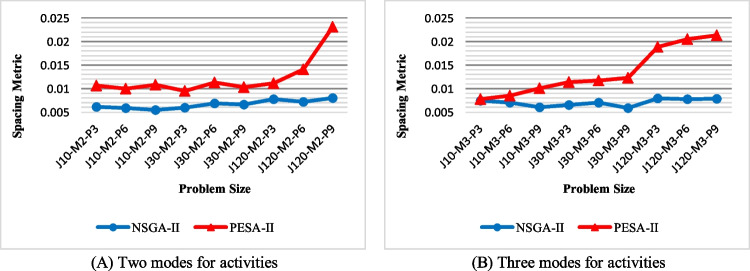
The efficiency of the algorithms with regard to SM

According to the results, none of the algorithms is absolutely superior to the other. PESA-II obtains higher quality solutions (higher QM and lower MID) than NSGA-II, contributing to better results for the decision-makers. On the other hand, NSGA-II obtains more diverse and regular solutions than PESA-II (higher NPS, higher DM (for medium and large problems), and lower SM). So, the solutions of NSGA-II give managers more flexibility in decision-making.

### Sensitivity analysis

Here, a sensitivity analysis was performed to investigate the impact of some parameters on the results of the presented MPSMOP model. In this regard, four parameters are selected, including the number of suppliers, number of modes, holding cost, and ordering cost. The analysis was performed on a problem that involved 3 projects, 3 modes, and 10 activities (J10-M3-P3). The results are discussed according to the average value of solutions in the determined Pareto optimal set.

**Fig. 19:**
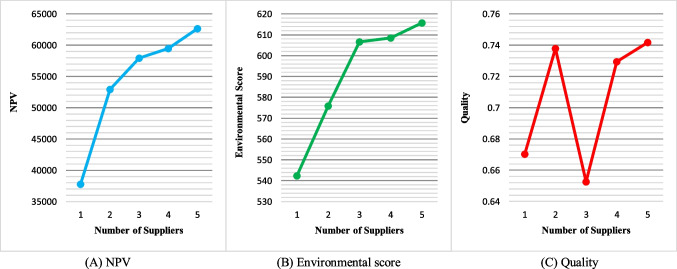
Sensitivity analysis of the objectives to the number of suppliers
- The number of modes: Fig. 20 represents the NPV, environmental score, and quality changes by varying the number of activity execution modes. Based on this figure, as the number of modes increases, NPV and quality values are improved due to the greater flexibility of the problem. However, the environmental score is almost indifferent to changes in this parameter.

- The number of suppliers: Fig. 19 represents the effect of changing the number of suppliers on the three objective functions, namely, NPV, environmental score, and quality, respectively. Based on this figure, the NPV and environmental score values are increased as the number of suppliers grows. That is because increasing the number of suppliers provides more flexibility for choices to make, leading to an improvement in terms of these objectives. From a practical point of view, the system’s economic savings and environmental score can be grown by increasing the number of potential suppliers (due to the suppliers’ competition and the difference in their selling price and environmental performance). This increase has a greater slope at the beginning. However, this parameter does not have an enormous impact on quality.

**Fig. 20:**
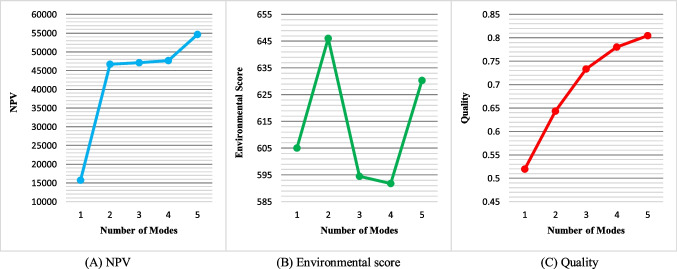
Sensitivity analysis of the objectives to the number of modes
- Holding and ordering costs: The effects of unit holding cost and ordering cost on NPV and discounted total cost are shown in Fig. 21. Based on this figure, as the holding and ordering costs increase, so do total costs, resulting in a decrease in NPV. The difference is that the decrease in NPV and the increase in total cost are more significant in-unit holding cost changes. In other words, holding cost is more sensitive than ordering cost from an economic perspective.

**Fig. 21:**
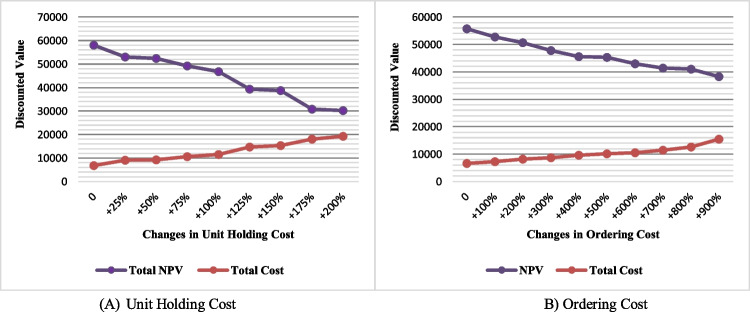
Sensitivity analysis of the NPV and discounted total cost to the unit holding and ordering costs

**Fig. 22:**
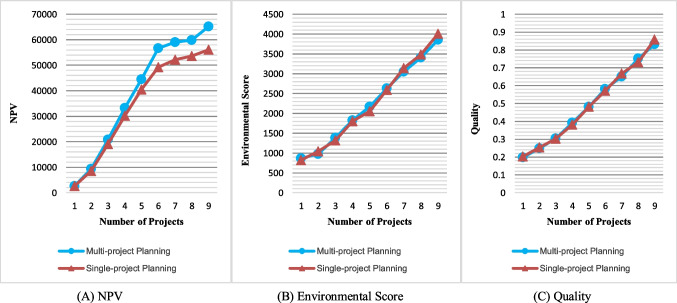
Comparing the results of single-project and multi-project planning

As mentioned earlier, investigating PSMOP for the multi-project environment is one of the main contributions of this work. Hence, the result of the multi-project model with that obtained by separately planning for each project was compared. This analysis was performed on the problem entitled J30-M3-P9 (1), which exists in the self-generated dataset. This problem is divided into nine sub-problems by considering different project numbers ranging from one to nine, each of which was examined in two cases of single planning of each project and multi-project planning. Figure 22 shows the results of this comparison for the three objectives. These results are the mean of Pareto optimal solutions obtained by the PESA-II.

This analysis shows that multi-project planning leads to the improvement and increase of NPV, and the more projects there are, the more significant this improvement will be. The savings from ordering costs and supplier discounts (when the required materials for the projects are ordered together) reduce costs and increase NPV. Therefore, significant cost savings can be made using the multi-project planning approach. However, multi-project planning does not differ in results from separate planning regarding environmental and quality objectives. In both planning cases, this is because an attempt is made to purchase materials from a supplier with better environmental performance and carry out activities in higher quality modes.

## Case study

**Fig. 23:**
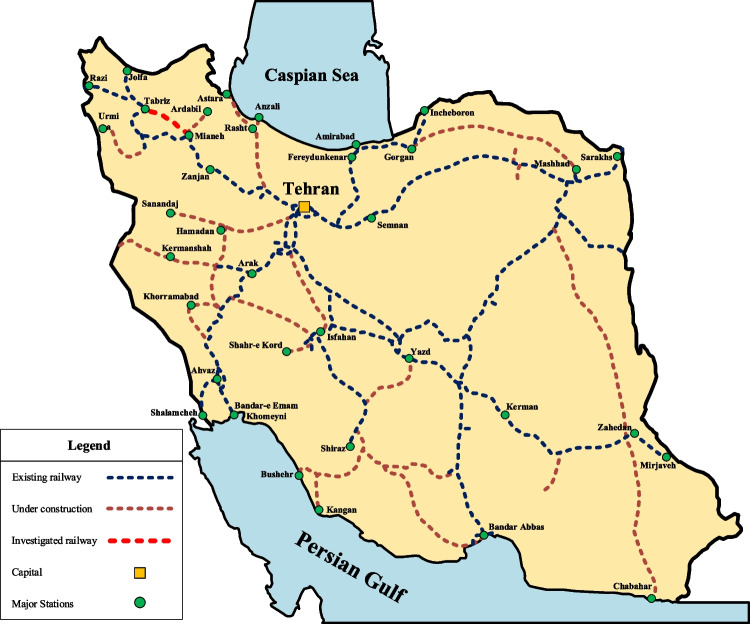
Railway system in Iran and location of implemented case study

As one of the critical elements of transit lines in developed societies, the railway system is considered an economical and safe mode of transportation. The Mianeh-Tabriz Railway is one of the main transportation projects in Iran that is under construction at the time of writing this paper. It is anticipated that this project will reduce the shipping distance between the capital (Tehran) and Tabriz by 5.5 h or 114 km. This project has a length of 183 km, is divided into 10 parts, and is located in the northwestern region of Iran (Fig. 23). During this project, 515 canals, 11 massive tunnels, 21 bridges with an average length of 0.38 km, and 10 galleries with an average length of 1.4 km have been built or are under construction.

In this paper, the presented MPSMOP model is implemented in the roadbed construction projects in parts 2 (Project 1) and 8 (Project 2) of this railroad (from km 21 to 30 and km 123 to 132). These two projects consist of 16 and 23 activities, respectively, whose implementation modes vary from 1 to 4 depending on the activity’s nature. The consumption resources are three types (cement, sand, and rebar), and the required renewable resources are thirteen types (specialized machinery and human resources). The key data and information about these projects are provided in Appendix 3.

### Model implementation

Based on Fig. 2, the steps for implementing the research framework in the case study are as follows:

- Calculating the environmental grades: the relevant and proper green criteria and metrics were selected for the system in the first stage. In this regard, the expert committee was provided with the list of environmental criteria and metrics represented in Table 2, and suitable indicators were selected through brainstorming and several meetings. In the following, Chang’s FAHP technique (Chang 1996) was utilized to calculate the weights of selected green criteria. According to this technique, the linguistic terms and explanations of the experts, such as very strong importance, moderate importance, and very weak importance, were used to make pairwise comparisons between the selected environmental criteria. Table 9 shows the selected criteria and metrics and their calculated weights.

**Table 9:** Details of environmental criteria and metrics

Criteria	Metrics	Weight
GHG emission	CO_2_, NO_2_, and CH_4_ emissions	0.304
Consumption of resource	Water and electricity consumption	0.256
Environmental management system (EMS)	EMS accomplishment and its quality	0.440

Three potential suppliers, namely, YNZ, ETP, and BMT, were identified from which the required materials can be purchased. Next, the data related to the selected green criteria and metrics were collocated. These data were mainly calculated based on the performance of the potential suppliers from the green perspective as follows.

For the first GHG emission criterion, three metrics, including the amount of CO_2_, NO_2_, and CH_4_ emissions, were measured. These metrics were estimated according to the type of vehicle used to transfer materials, the distance between construction sites, and the location of suppliers. The trucks’ standards and pollution level per distance were extracted from Jonidi Jafari and Arfaeinia (2016).

In terms of resource consumption, two water and electricity consumption metrics were considered to be measured. Values of these metrics depend on each supplier’s production system, and they were estimated based on the production capacity, machinery, and production process devoted to each one.

To measure the EMS accomplishment and its quality, three levels were defined: not satisfied, satisfied but not authenticated, and satisfied with authentication. In the next step, suppliers are categorized into these three levels so that the suppliers who do not have any documentation are placed in the first category. Those who had required documentation but did not have a valid certificate (for example, ISO 14000) were categorized into the second level. The third category was also assigned to suppliers with valid EMS certificates.

Next, the FIS was utilized to determine the environmental grade of suppliers. The scores were specified by defining the membership function for input and output values and using the fuzzy logic toolbox in MATLAB. The environmental grades of suppliers in each criterion are shown in Table 10. In the last row of this table, the environmental scores of suppliers are given, which are calculated using weighted averaging.

**Table 10:** Environmental grades of suppliers

Criteria	Weight	Scores of suppliers		
		YNZ	ETP	BMT
GHG emission	0.304	0.558	0.937	0.162
Consumption of resource	0.256	0.278	0.063	0.899
Environmental management system (EMS)	0.440	0.25	0.75	0.75
Total score		**0.351**	**0.631**	**0.609**

As can be seen, the ETP could achieve the highest score (0.631) due to his better environmental performance. We refer the readers to the case study of Habibi et al. (2019) for more detailed information about this step.

- Quantifying the quality of activity modes: as mentioned before, the CONQUAS technique is used to estimate the quality values for each mode of activity. Based on the steps in the “Quantifying the quality of activities” section, at first, different elements and standards of each item (activity) were extracted from Low and Ong (2014). For example, the elements and standards for formwork operation are summarized in Table 11.

**Table 11:** The items and standards for formwork operation

Activity	Item	Standard
Formwork operation	(a) Formwork dimensions and openings for services	1. Tolerance for cross-sectional dimensions of cast in situ and precast elements: + 10 mm/ − 5 mm
		2. Tolerance for penetration/opening for services: + 10 mm for size and ± 25 mm for location
		3. Tolerance for length of precast members: • Lower than 3 m: ± 6 mm • Between 3 m and 4.5 m: ± 9 mm • Between 4.5 m and 6 m: ± 12 mm • Higher than 6 m: ± 6 mm
	(b) Alignment, plumb, and level	1. Tolerance for the departure of any point from its position: 10 mm
		2. Tolerance for plumb: 3 mm/m, max 20 mm
		3. Maximum deviation of the mean level of staircase thread to temporary benchmark: ± 5 mm
		4. For cast-in situ elements, the deviation of level of any point from the intended level: ± 10 mm
	(c) Condition of formwork, props, and bracing	1. Formwork should be free from faults
		2. Before concreting, the interior should be free from residue
		3. All formwork joints should not have gaps to hamper leaking
		4. There should be adequate support, bracing, and tie-back for the formwork to prevent bulging or displacement of structural elements

Then, the quality grade of activities in similar implemented projects was determined to estimate the expected quality of existing modes. So, the experts were asked to evaluate each mode of activity in existing projects based on the implemented items in parts 1 and 5 of the Mianeh-Tabriz Railway Project, which had already been completed. For each mode, if all requirements were fulfilled, then the quality of that mode would be equal to 100%. Otherwise, the fulfilled requirements ratio was considered the value of quality. The calculated quality values are provided in Appendix 3.

**Fig. 24:**
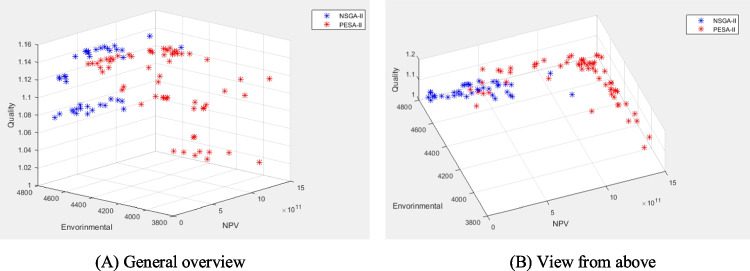
Pareto optimal front from two perspectives

- Building the mathematical model and achieving solutions: in this stage, the collected information and calculated data were used as the input for the proposed multi-objective MILP model. Moreover, both metaheuristics (NSGA-II and PESA-II) were used to solve the model. Figure 24 represents the outcome and the Pareto optimal front from two perspectives. As can be seen, the Pareto front is spread out like a curved plane in the objective space. So, decision-makers can select one of these solutions based on their utilities.

**Fig. 25:**
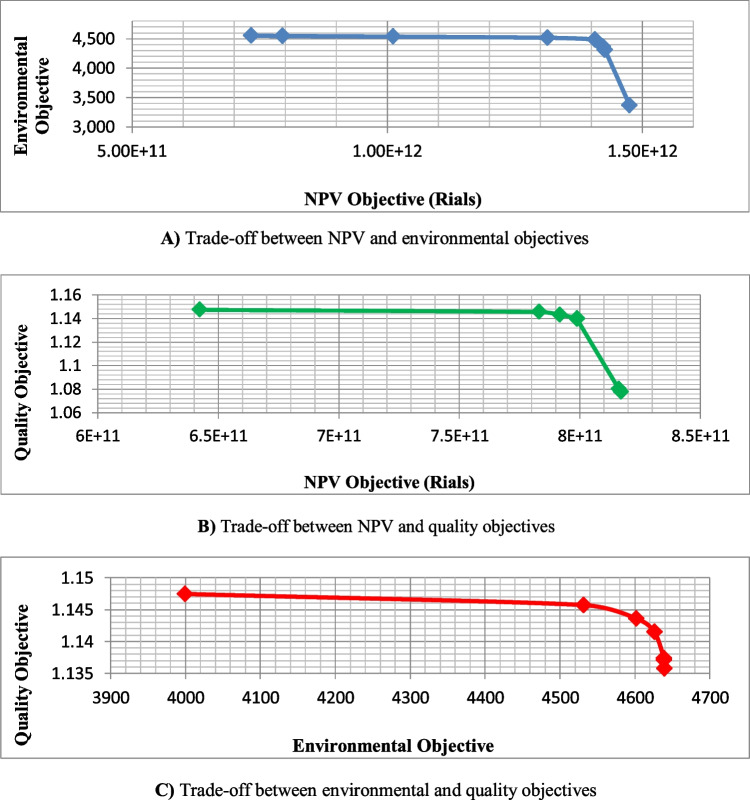
The decision maps for dual combinations of objectives

The trade-off between the three objectives was examined to analyze the solutions more closely. In this regard, the case study data were utilized to solve the problem with the various dual combinations of objectives. Figure 25 explains the trade-offs among NPV, environmental, and quality objective functions. Based on this figure, the trade-off rate for NPV and environmental objectives changes significantly when $1.38E+12<NPV<1.42E+12$. If $NPV<1.38E+12$, the decision-maker can decrease NPV with a minimal increase in environmental score. In contrast $NPV>1.42E+12$, a slight additional increase in NPV contributes to a substantial decrease in environmental score. A similar trend is observed for the trade-off between NPV/quality and environmental/quality objectives.

The other point is solutions with a low environmental score, low quality, and high NPV. These solutions are mainly devoted to decisions based on which inexpensive and low-quality modes of activities are selected. Although inexpensive modes lead to increasing NPV, they consume fewer resources, and as a result, the environmental impact decreases (because fewer materials are purchased and the pollution from the production process and transportation is reduced). In addition, low-quality modes of activities contribute to decreasing the quality objective. It is observed that these solutions are found by PESA-II (not by NSGA-II). As mentioned earlier, PESA-II can determine better diversified optimal solutions than NSGA-II in some cases (Gadhvi et al. 2016). In other words, PESA-II may explore some parts of the solution space that NSGA-II did not search. This is precisely what can be observed in this figure.

In order to exemplify the MPSMOP model, among the optimal Pareto solutions of Fig. 24, a solution is selected, which leads to a good performance in terms of environmental score and project completion time (Cmax) criteria. This solution has values of 1.493E + 12, 4653.31 and 1.12, respectively, in NPV, environmental, and quality objective functions. Based on this solution, the optimal decisions for Project 2 (part 8 of the railway system) are summarized in Table 12.

**Table 12:** An example of the MPSMOP model

Activity	Mode	Start time	Finish time	Ordering time			Supplier		
				*f*_1_	*f*_2_	*f*_3_	*f*_1_	*f*_2_	*f*_3_
1	1	0	0	–*	–	–	–	–	–
2	1	0	100	–	–	–	–	–	–
3	1	100	113	–	–	–	–	–	–
4	2	113	238	–	–	–	–	–	–
5	3	238	407	–	–	–	–	–	–
6	1	407	502	–	–	–	–	–	–
7	1	113	235	–	–	–	–	–	–
8	1	502	503	–	–	–	–	–	–
9	1	503	504	–	–	–	–	–	–
10	1	504	509	499	490	–	2	1	–
11	1	509	535	–	–	498	–	–	2
12	2	535	580	–	–	–	–	–	–
13	3	580	635	570	565	562	2	2	3
14	1	635	636	–	–	–	–	–	–
15	1	636	637	–	–	–	–	–	–
16	1	637	638	–	–	–	–	–	–
17	1	638	643	630	632	–	2	3	–
18	1	643	666	–	–	632	–	–	2
19	2	666	711	–	–	–	–	–	–
20	1	711	738	630	701	–	2	2	–
21	1	738	739	–	–	–	–	–	–
22	4	739	779	–	–	–	–	–	–
23	1	779	779	–	–	–	–	–	–

*This means that the activity does not require any non-renewable resources (materials)

As the solution has the minimum Cmax, it can be seen that the start time of activities is equal to the maximum finish time of its predecessor activities. In other words, there is no buffer time between the finish time of one task and the start time of its following task in the critical path. Besides, the ordering time of materials for each activity is before the activity’s start time, with a negligible time gap due to the supply lead time. It can also be seen that the orders of material type 1 (*f*_1_) related to activities 17 and 19 are ordered at the same time (630) and from the same supplier (supplier 2) to take advantage of the discount benefits. However, for other activities, the required materials are ordered individually. From the environmental point of view, the second and third suppliers with the highest environmental scores (0.631 and 0.609) are frequently selected. For this reason, the solution is appropriate in terms of environmental benefits. Hence, using the MPSMOP model enables the project management team to make precise project scheduling and material ordering decisions in an integrated manner to achieve optimal economic, environmental, and quality performance.

### Quality of solutions

After all analyses, there is still an open question to be answered: “how reliable are the obtained solutions by proposed metaheuristics?” This subsection scrutinizes the quality of obtained solutions compared to the exact solution. In this regard, the mathematical model was solved using the weighted sum method (Habibi et al. 2017a) and the case study data. The weighted sum method is one of the most common techniques to solve multi-objective optimization problems. It explores solution space by aggregating the objectives into a single scalar function and devoting a weight to each. Altering the weights of objectives results in finding different solutions from the Pareto optimal front. The problem of the case study was coded in GAMS 24.0.1. We also used various weights randomly generated to maintain the trade-off among objectives. Table 13 represents the weights, the obtained solutions by the CPLEX solver, and the computational time to solve each problem.

**Table 13:** Results of applying the weighted sum method

Row	Weights			Solution			Computational time (h:m:s)
	First objective (NPV)	Second objective (environmental)	Third objective (quality)	NPV	Environmental	Quality**	
1	0.2	0.4	0.4	8.9136E + 11	4657.6	1.1345	00:03:22
2	0.3	0.4	0.3	1.3121E + 12	4495.6	1.1262	00:04:52
3	0.05	0.35	0.6	2.6689E + 11	4426.1	1.1471	00:07:56
4	0.85	0.1	0.05	1.4331E + 12	3991.9	1.0168	00:03:07
5	0.6	0.1	0.3	1.4257E + 12	4245.8	1.0892	00:02:17
6	0.2	0.45	0.35	7.0949E + 11	4687.7	1.1193	00:17:55
7	0.45	0.35	0.2	1.4294E + 12	4474.7	1.0613	00:02:40
8*	0.1	0.4	0.5	2.6689E + 11	4426.1	1.1471	00:02:27
9	0.6	0.3	0.1	1.4329E + 12	4551.1	1.0090	00:02:31
10*	0.15	0.55	0.3	7.0949E + 11	4687.7	1.1193	00:06:28
11*	0.5	0.2	0.3	1.4257E + 12	4245.8	1.0892	00:02:25
12*	0.8	0.1	0.1	1.4331E + 12	3991.9	1.0168	00:02:18
13	0.25	0.45	0.3	9.2858E + 11	4717.1	1.0874	00:02:47
14	0.15	0.45	0.4	6.9613E + 11	4689.0	1.1350	00:02:39
15*	0.4	0.25	0.35	1.3121E + 12	4495.6	1.1262	00:02:23
Total computational time							**01:06:07**

*Duplicated solution

**To have a measure between 0 and 1 for quality, values can be divided by the ideal point (*P* = 2)

Table 13 shows that only ten unique solutions from the Pareto optimal front were found, although fifteen different weights were used. Hence, this methodology may include repeated attempts, each of which can take considerable computational time. This limitation is not significant in metaheuristics, where the process saves time and leads to higher speed for exploring Pareto optimal front. Here, the total computational time the weighted sum method devotes to finding duplicated solutions was about 16 min. It equals almost 24% of the total computational time.

**Fig. 26:**
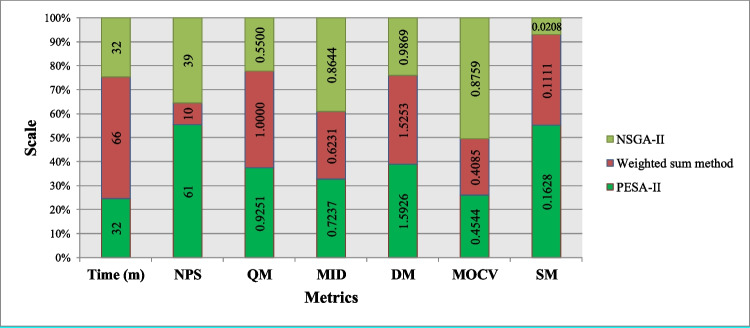
Comparing the performance of three solution methods

The solutions obtained by NSGA-II and PESA-II were compared to those of the exact method whose results are reported in Fig. 26.

- Computational time: metaheuristic algorithms obtained the solutions in 32 min, while the weighted sum method allocated twice as much as this time. Obviously, exploring good quality solutions within a reasonable amount of time is the advantage of metaheuristics compared to the exact solution techniques.
- NPS: although the weighted sum method had more computational time than metaheuristics, the number of Pareto solutions determined by this method is considerably less than those obtained by metaheuristics. PESA-II and NSGA-II explored 61 and 39 unique solutions, while the weighted sum method explored only 10.
- QM: regarding the quality, it is no wonder that metaheuristics cannot dominate the solutions obtained by the weighted sum method. However, 45% of solutions found by NSGA-II are dominated by either the PESA-II or the exact method. This value is only 7.5% when we refer to the PESA-II, which proves the high quality of solutions found by PESA-II. Considering the 32-min computational time and 61 Pareto solutions (compared to the exact method), 92.5% reliability seems acceptable.
- MID: this metric shows that the solutions found by the weighted sum method were closer to the ideal solution, and the solutions obtained by PESA-II and NSGA-II come next from this point of view. This result confirms the results concluded from QM.
- DM: in terms of diversity, solutions of PESA-II have the most, and those of the weighted sum method and NSGA-II come next in order. It means that PESA-II provides the decision-makers with more flexibility and power of choice. However, solutions of the weighted sum method do not differ much from those of PESA-II. Solving the problem with a higher number of diverse weights can help improve this index in the weighted sum method, but this, in turn, will also increase the computational time. The superiority of PESA-II compared to NSGA-II in DM is also obvious in Fig. 24.
- MOCV: considering the solutions’ quality and diversification, MOCV represents the superiority of the weighted sum method, where PESA-II and NSGA-II come next in order. Although PESA-II acted better than the weighted sum method in terms of diversity, the considerably better quality of solutions obtained by the weighted sum method led to better performance of this technique in terms of MOCV.
- SM: this criterion represents the greater regularity in solutions obtained by NSGA-II compared to the weighted sum method and PESA-II. It should be noted that increasing the number of populations in metaheuristics and the number of weights in the weighted sum method can provide higher regularity of solutions.

## Conclusion

The multi-project scheduling and material ordering problem (MPSMOP) is considered a specific form of the project scheduling problem. Unlike conventional project planning, this approach maintains a proper trade-off between scheduling and material procurement expenses to help project managers make more accurate decisions. Undoubtedly, the importance of this trade-off is more profound in the multi-project scheduling environment. In this study, a tri-objective MILP model was proposed to determine the scheduling and ordering decisions so that the NPV, environmental score, and total quality of implemented projects are maximized simultaneously. The scheduling was studied in a multi-project environment with multi-mode activities where the required materials were ordered from multi-suppliers. This paper first presented a comprehensive overview of the MPSMOP, discussed the related literature, and expressed the contributions of existing research. Since the proposed model came into the category of NP-hard problems, two powerful metaheuristic solution techniques, entitled NSGA-II and PESA-II, were customized to solve the problem. After the parameter tuning process through the Taguchi design of experiments, the performance of both solution methods was evaluated on several self-generated datasets. The results showed that although PESA-II produces high-quality solutions, NSGA-II can increase decision-making flexibility by providing a higher number of Pareto solutions and more diverse and regular frontiers on average. Eventually, the designed framework was implemented in a case study on railway construction projects in parts 2 and 8 of the Mianeh-Tabriz transit line in Iran. The results obtained from the case study verified the performance of the presented MPSMOP model and showed that it enables the project management team to make more precise decisions. For future research, it is suggested that some other realistic assumptions be added to the problem. For example, resource pool for materials, sharable renewable resources among projects, and preemptive activities have great potential for investigation. It is also suggested that new mechanisms for coding and decoding be investigated, and then, the results will be compared with the procedure presented in this paper. One of the main issues in the material ordering problem is supply uncertainty which can be addressed by future research.

## Data Availability

The self-generated datasets can be obtained from the following link: https://www.unsw.adfa.edu.au/dsar-group/dsarg-datasets.

## Appendix 1

In the following, the fuzzy AHP method based on Chang (1993) analysis approach is explained:

- Step 1—creating the hierarchical model: at first, the criteria, sub-criteria, and alternatives should be defined. Then, a hierarchical model is identified to clear the model’s elements.
  Step 2—pairwise comparisons based on the defined fuzzy scale: in this step, pairwise comparisons should be carried out based on the fuzzy scale shown in Table 14.
  **Table 14:** The fuzzy scale used in this research
  Lingual variable	Fuzzy scale
  Equal importance	(1, 1, 1)
  Very weak advantage	(0.5, 1, 1.5)
  Weak advantage	(1, 1.5, 2)
  Medium advantage	(1.5, 2, 2.5)
  Strong advantage	(2, 2.5, 3)
  Very strong advantage	(2.5, 3, 3.5)
- Step 3—integration of pairwise comparisons: when several experts respond to pairwise comparisons, the geometric mean method is used to integrate them and obtain an integrated paired comparison matrix. In integrating fuzzy matrices, we get the geometry of first, second, and third values separately.
- Step 4—calculation of weights using the analysis method: first, the fuzzy numbers of *S*_*i*_ for each row of the fuzzy pairwise comparison matrix are obtained based on Eq. (27):
  27 $$S_i=\sum \limits_{j=1}^mM_{gi}^j\otimes\left[\sum \limits_{i=1}^n\sum \limits_{j=1}^mM_{gi}^j\right]^{-1}$$
  where *g*_*i*_ is the target set and ${M}_{gi}^{j}$ is triangular fuzzy number; then, the degree of preference for each *S*_*i*_ over *S*_*k*_ should be obtained based on Eq. (28).
  28 $$V\left(S_i>S_k\right)=\left\{\begin{array}{c}1\\0\\ \frac{l_k-u_i}{\left(m_i-u_i\right)-\left(m_k-l_k\right)}\end{array}\begin{array}{c}m_i\geq m_k\\l_k\geq u_i\\ \mathrm{Otherwise}\end{array}\right.$$

In the last step, the weights are calculated using Eq. (29).

29 $$V\left(S\geq S_1,S_2,\dots,S_k\right)=minV\left(S\geq S_i\right)\;i=1,2,\dots,k$$

## Appendix 2

Table 15

**Table 15:** General guideline for using datasets

Subject		Descriptions	Example
File title		The name of each file is listed in the JW-MX-PY (Z) format, where W, X, Y, and Z indicate the number of activities, the number of modes, the number of projects, and the problem number, respectively	For example, the file entitled J10-M3-P3 (1) devotes to the first dataset, including 3 projects with 10 activities and 3 execution modes
Contents (sheets)	Successor list	In the first sheet entitled “Successors List,” the precedence relationships between activities are defined	As shown in the Successors List Section of J10-M3-P3 (1), activities 9 and 10 are the successor activities for activity 7 and also, activity 3 is a precedence activity for activities 10 and 11
	Duration	This sheet expresses the processing time of different modes of activities related to each project	As shown in the Duration Section of J10-M3-P3 (1), the processing time of activity 7 in mode 3 for Project 2 is equal to 10
	Deadline	This sheet belongs to the due dates of completing the projects	Based on J10-M3-P3 (1), the due dates of projects 1, 2, and 3 are 24, 22, and 20, respectively
	Resource usage	This section indicates the number of renewable resources that are required for performing activities of different projects	The number of resource type 4 for doing activity 7 in mode 3 for Project 2 is equal to 7 in J10-M3-P3 (1)
	Rmax	The Rmax Section shows the number of available renewable resources in each project site	In J10-M3-P3 (1), there are 16 and 25 units for resource types 2 and 3 in Project site 1
	Material usage	The Material Usage Section expresses the amount of material needed for performing activities of projects in different modes	In J10-M3-P3 (1), 8 and 6 units of material type 1 and 2 are needed to perform activity 7 with mode 2 in Project 3
	Ordering cost	In this section, the ordering cost of each material type from each supplier is provided	As can be seen in J10-M3-P3 (1), the ordering cost of material type 3 from supplier 2 is equal to 10
	Holding cost	In this sheet, the holding cost of each material type in each period per unit is given	The holding cost of material type 3 is equal to 4 in J10-M3-P3 (1)
	Penalty-bonus	This part includes the bonus/penalty cost due to completing the projects before/after the pre-determined due dates per each period	In J10-M3-P3 (1), the bonus and penalty cost for project 3 is equal to 18 and 13, respectively
	CF +	The cash inflow of performing activities is provided in this section	The cash inflow of activity 7 of Project 2 is 5343
	CF −	The cash outflow of performing activities is provided in this section	The cash outflow of activity 7 of Project 2 in mode 3 is 68
	Lead time	In this part, the lead time for purchasing all material type from suppliers for all projects is defined	As shown in the Lead Time Section of J10-M3-P3 (1), purchasing material type 2 from supplier 3 for Project 1 is equal to 6
	Ir-w	This sheet provides the interest rate and importance weight between the average and minimum qualities	As shown in J10-M3-P3 (1), the interest rate and the importance weight are 0.064 and 0.37, respectively
	Purchase cost	This sheet belongs to the unit procurement cost of materials ordered from each supplier with discount policy consideration	Based on J10-M3-P3 (1), the purchase cost of material type 1 from supplier 3 is 6 and 3 for the first and second discount ranges, respectively
	Discount intervals	This section indicates the limitation on discount intervals for all material types related to each supplier	The third supplier has two discount intervals for material type 2, [0, 12] and (12, infinity), in J10-M3-P3 (1). In other words, supplier 3 offers a lower price for purchases of more than 12 units
	Environmental grades	The environmental Grades Section shows the score of each supplier from an environmental perspective	In J10-M3-P3 (1), the environmental grade of the second supplier for supplying material types 1 and 2 is 0.726 and 0.557, respectively
	Quality	The Quality Section expresses the quality of performing activities in different modes	In J10-M3-P3 (1), activity 7 in Project 1 can be executed in three modes with quality values 0.973, 0.585, and 0.677

## Appendix 3

Table 16

**Table 16:** Data of the projects in the case study

Project	Plan	Num	Activity	Duration (days)				Quality				Predecessor
				Mode 1	Mode 2	Mode 3	Mode 4	Mode 1	Mode 2	Mode 3	Mode 4	
2^nd^ part of trackbed construction	Initial activities	1	Start	0	–	–	–	–	–	–	–	–
	Equipping process	2	Ground provision and workshop equipment	100	140	–	–	0.90	0.76	–	–	1
	Land preparation	3	Landscaping and building the access road	13	–	–	–	1	–	–	–	2
	Earthwork operations (km 21–30)	4	Clearing the land	100	125	150	175	0.72	1	0.79	0.83	2, 3
		5	Route embankment	113	137	169	172	0.73	0.80	0.83	0.62	3, 4
		6	Route consolidation	95	130	146	177	0.80	0.81	0.71	0.77	5
		7	Route excavation	122	195	274	326	0.87	0.90	0.65	0.87	3
	Construction of bridge No. 1 (at km 28)	8	Map approval	1	–	–	–	1	–	–	–	2, 6, 7
		9	Surveying and map controlling	1	–	–	–	1	–	–	–	8
		10	Digging up process	5	–	–	–	1	–	–	–	9
		11	Reinforcement process	26	38	46	63	0.90	0.83	0.62	0.84	10
		12	Concrete Shuttering	36	45	68	81	0.80	0.94	0.82	0.74	11
		13	Formwork operations	30	39	55	75	0.81	0.72	0.90	1	12
		14	Backfilling process	1	–	–	–	1	–	–	–	13
	Finishing activities	15	Cleaning up the workshop	10	20	30	40	0.74	0.88	0.93	0.95	2, 3, 6, 7, 14
		16	Finish	0	–	–	–	–	–	–	–	15
8^th^ part of trackbed construction	Initial activities	1	Start	0	–	–	–	–	–	–	–	–
	Equipping process	2	Ground provision and workshop equipment	100	140	–	–	0.90	0.76	–	–	1
	Land preparation	3	Landscaping and building the access road	13	–	–	–	1	–	–	–	2
	Earthwork operations (km 123–132)	4	Clearing the land	100	125	150	175	0.72	1	0.79	0.83	2, 3
		5	Route embankment	113	137	169	172	0.73	0.80	0.83	0.62	3, 4
		6	Route consolidation	95	130	146	177	0.80	0.81	0.71	0.77	5
		7	Route excavation	122	195	274	326	0.87	0.90	0.65	0.87	3
	Construction of Bridge No. 1 (at km 127)	8	Map approval	1	–	–	–	1	–	–	–	2, 6, 7
		9	Surveying and map controlling	1	–	–	–	1	–	–	–	8
		10	Digging up process	5	–	–	–	1	–	–	–	9
		11	Reinforcement process	26	38	46	63	0.90	0.83	0.62	0.84	10
		12	Concrete Shuttering	36	45	68	81	0.80	0.94	0.82	0.74	11
		13	Formwork operations	30	39	55	75	0.81	0.72	0.90	1	12
		14	Backfilling process	1	–	–	–	1	–	–	–	13
	Construction of bridge No. 2 (at km 130)	15	Map approval	1	–	–	–	1	–	–	–	2, 6, 7, 14
		16	Surveying and map controlling	1	–	–	–	1	–	–	–	15
		17	Digging up process	5	–	–	–	1	–	–	–	16
		18	Reinforcement process	23	33	44	60	0.90	0.82	0.74	0.93	17
		19	Concrete Shuttering	34	45	62	77	0.81	0.86	0.79	0.62	18
		20	Formwork operations	27	36	52	71	0.81	0.72	0.90	1	19
		21	Backfilling process	1	–	–	–	1	–	–	–	20
	Finishing activities	22	Cleaning up the workshop	10	20	30	40	0.74	0.88	0.93	0.95	2, 3, 6, 7, 21
		23	Finish	0	–	–	–	–	–	–	–	22

## Footnotes

1 https://www.bca.gov.sg/Professionals/IQUAS/others/CONQUAS8.pdf
